# Phytochemical Constituents from *Ardisia sylvestris* Pitard Leaves: Enzyme Inhibition, Antioxidant, and Antimicrobial Activities with Molecular Simulation Insights

**DOI:** 10.3390/cimb48100986

**Published:** 2026-09-24

**Authors:** Nguyen Thi Thu Ha, Hai Pham-The, Dao Long Vu, Nguyen Van Tuyen, Phan Thu Huyen, Nguyen Thi Thuy Linh, Bui Hai Ninh, Le Thi Tu Anh, Ba Thi Cham, Nguyen Thanh Tra, Nguyen Thi Van Anh, Bakhtiyor Rasulev, Gerardo M. Casanola-Martin

**Affiliations:** 1 Institute of Chemistry, Vietnam Academy of Science and Technology (VAST), 18 Hoang Quoc Viet, Nghia Do, Hanoi 10000, Vietnam; thuha.vast@gmail.com (N.T.T.H.); ngvtuyen@hotmail.com (N.V.T.); thuylinh1992.bk@gmail.com (N.T.T.L.); lntuanh@gmail.com (L.T.T.A.); chamhoasinh@gmail.com (B.T.C.); nguyenthanhtravast@gmail.com (N.T.T.); 2 Department of Chemistry, Graduate University of Science and Technology, VAST, 18 Hoang Quoc Viet, Nghia Do, Hanoi 10000, Vietnam; phanhuyen179@gmail.com; 3 Department of Medicine and Pharmacy, University of Science and Technology of Hanoi (USTH), VAST, 18 Hoang Quoc Viet, Nghia Do, Hanoi 10000, Vietnam; hpham.phd@gmail.com (H.P.-T.); vulong0512@gmail.com (D.L.V.); nguyen-thi-van.anh@usth.edu.vn (N.T.V.A.); 4 Department of Medicinal Chemistry and Drug Quality Control, Hai Phong University of Medicine and Pharmacy, 72A Nguyen Binh Khiem, Gia Vien, Hai Phong City 18000, Vietnam; ninhdyhp@gmail.com; 5 Department of Coatings and Polymeric Materials, North Dakota State University, Fargo, ND 58102, USA; bakhtiyor.rasulev@ndsu.edu

**Keywords:** *Ardisia sylvestris*, α-glucosidase inhibition, acetylcholinesterase inhibition, antioxidant activity, antimicrobial activity, target prioritization, in vitro–in silico approaches

## Abstract

A phytochemical investigation of the ethanolic extract of *Ardisia sylvestris* Pitard leaves led to the isolation and identification of eight compounds, including 3S,5R,6R,9S-tetrahydroxymegastigman-7-ene (**1**), myricetin-3-*O*-rutinoside (**2**), phenyl ethyl-*O*-α-L-rhamnopyranosyl-(1→2)-β-D-glucopyranoside (**3**), 2-*O*-(trans-coumaroyl)malic acid 1-methyl ester (**4**), adenosine (**5**), gallic acid (**6**), 5-(8Z-heptadecenyl)resorcinol (**7**), and mandelic acid (**8**). Their structures were identified by NMR analysis, supported by MS data where available and comparison with published spectroscopic data. The isolated compounds were evaluated for α-glucosidase inhibitory, acetylcholinesterase (AChE) inhibitory, antioxidant, and antimicrobial activities. Among them, compound **7** showed the most promising biological profile, showing potent α-glucosidase inhibition (IC_50_ = 7.56 μg/mL), markedly stronger than acarbose (IC_50_ = 151.09 μg/mL), together with moderate AChE inhibitory activity (IC_50_ = 14.75 μg/mL). Compound **7** also displayed strong DPPH radical-scavenging activity (IC_50_ = 10.04 μg/mL), comparable to quercetin, and moderate antibacterial activity against *Staphylococcus aureus* (MIC = 64 μg/mL). Molecular docking and molecular dynamics simulations revealed favorable binding modes and stable interactions of compound **7** within the active sites of α-glucosidase and AChE. For the antibacterial activity, substrate-similarity-based target prioritization followed by comparative docking highlighted FabI as the highest-priority candidate target for compound **7**. These findings identify 5-(8Z-heptadecenyl)resorcinol as a multifunctional bioactive constituent of *A. sylvestris* and provide complementary structural insights into its interactions with α-glucosidase and acetylcholinesterase.

## 1. Introduction

The genus *Ardisia* (Primulaceae), comprising approximately 500 species distributed throughout tropical and subtropical regions, is recognized as a rich source of structurally diverse secondary metabolites, including triterpenoid saponins, flavonoids, quinones, phenolic acids, bergenin derivatives, and alkylresorcinols [1,2,3]. Numerous *Ardisia* species have been reported to possess antioxidant, antimicrobial, anti-inflammatory, antidiabetic, neuroprotective, and anticancer activities, highlighting their pharmacological potential [4,5,6]. Nevertheless, many members of the genus remain insufficiently investigated, particularly with respect to the relationship between their phytochemical composition and biological activities.

*Ardisia sylvestris* Pitard, commonly known in Vietnam as “Khôi tía” or “Khôi nhung”, is traditionally used to treat gastrointestinal disorders and inflammatory conditions [7]. Previous studies have mainly focused on crude extracts, reporting antioxidant and anti-inflammatory activities together with preliminary phytochemical information [8]. However, direct evidence linking individually isolated constituents of *A. sylvestris* to specific biological activities remains limited. Establishing such relationships is essential for understanding the pharmacological basis of this medicinal plant and for identifying its principal bioactive constituents.

The integration of phytochemical investigation with biological evaluation and molecular simulation has become an effective strategy for characterizing natural products and elucidating their mechanisms of action [9,10,11]. While in vitro assays provide direct evidence of biological activity, in silico simulation, such as molecular docking and molecular dynamics, offer complementary insights into protein–ligand interactions and binding stability, thereby facilitating the interpretation of experimental observations [12,13].

Despite previous reports on the phytochemical composition and biological activities of crude extracts of *A. sylvestris*, the contribution of individual constituents to its enzyme-inhibitory, antioxidant, and antimicrobial effects remains poorly defined. In particular, compound-level evidence linking isolated metabolites with α-glucosidase and acetylcholinesterase inhibition has been limited, and the molecular basis of these activities has not been systematically explored. Therefore, the present study aimed to isolate and structurally characterize constituents from *A. sylvestris* leaves, evaluate their α-glucosidase inhibitory, acetylcholinesterase inhibitory, antioxidant, and antimicrobial activities, and identify the principal bioactive constituent. Molecular docking and molecular dynamics simulations were subsequently employed to provide complementary mechanistic insights into the experimentally observed activities.

## 2. Materials and Methods

### 2.1. Plant Material

Leaves of *Ardisia sylvestris* Pitard were collected in Tam Duong district, Lai Chau Province, Vietnam, in June 2023. The plant material was authenticated by Associate Professor Vu Tien Chinh (Vietnam National Museum of Nature, VAST). A voucher specimen (AS-2023/LC) was deposited at the Institute of Chemistry, Vietnam Academy of Science and Technology (VAST), Hanoi, Vietnam.

### 2.2. General Experimental Procedures

NMR spectra were recorded on a Bruker Avance 600 MHz spectrometer (Bruker BioSpin, Rheinstetten, Germany), operating at 600 MHz for ^1^H and 150 MHz for ^13^C nuclei. High-resolution ESI-MS spectra were obtained using a Thermo Scientific LTQ Orbitrap XL mass spectrometer (Thermo Fisher Scientific, Waltham, MA, USA). Column chromatography was performed using silica gel (40–63 μm), Sephadex LH-20, Diaion HP-20, and RP-18 materials (Merck, Darmstadt, Germany). Analytical and preparative thin-layer chromatography (TLC) were carried out on silica gel 60 F254 plates (Merck, Darmstadt, Germany).

### 2.3. Extraction, Isolation and Structural Identification

A schematic overview of the extraction, fractionation, and isolation procedures is provided in Figure S1. A similar ethanolic extraction approach has previously been reported for *A. sylvestris* leaves [7,8]. Fresh leaves of *A. sylvestris* (30 kg) were air-dried and ground to obtain 5.0 kg of powdered material. The powdered leaves were exhaustively extracted three times with 96% ethanol at 45–50 °C for 24 h per extraction cycle. The combined ethanol extracts were filtered and concentrated under reduced pressure. The resulting crude extract was suspended in warm water and successively partitioned with n-hexane and ethyl acetate to afford the n-hexane (50 g), EtOAc (187 g), and aqueous (500 g) fractions.

The EtOAc fraction (40 g) was subjected to silica gel column chromatography and eluted with an EtOAc–MeOH gradient system (100:0 to 0:100, *v*/*v*) to obtain five fractions (E1–E5). Fraction E1 (5 g) was further separated by silica gel column chromatography using CH_2_Cl_2_–MeOH with increasing MeOH content from 5% to 30%, yielding eight subfractions (E1.1–E1.8). Subfraction E1.4 (30 mg) was purified by silica gel column chromatography using n-hexane–acetone (2:1, *v*/*v*) to obtain compound **7** (6 mg). Fraction E5 (6.5 g) was chromatographed on a Sephadex LH-20 column eluted with CH_2_Cl_2_–MeOH (1:9, *v*/*v*) to obtain five subfractions (E5.1–E5.5). Subfraction E5.3 (120 mg) was further purified on Sephadex LH-20 using CH_2_Cl_2_–MeOH (1:9, *v*/*v*) to yield three subfractions (E5.3.1–E5.3.3). Subfraction E5.3.2 was purified by preparative TLC to obtain compound **1** (5 mg).

The AqExt fraction (100 g) was subjected to Diaion HP-20 column chromatography and eluted with a MeOH–H_2_O gradient system to obtain four fractions: B1 (100% H_2_O), B2 (70% H_2_O), B3 (50% MeOH), and B4 (100% MeOH). Fraction B3 (240 mg) was chromatographed on a Sephadex LH-20 column eluted with MeOH to afford seven subfractions (B3.1–B3.7). Subfraction B3.2 (50 mg) was re-purified on Sephadex LH-20 using MeOH to yield compound **5**, adenosine (9 mg). Subfraction B3.6 (190 mg) was purified by RP-18 reversed-phase column chromatography using MeOH–H_2_O (1:1, *v*/*v*) to obtain five subfractions (B3.6.1–B3.6.5). Subfraction B3.6.3 (60 mg) was re-purified on Sephadex LH-20 using MeOH to yield compound **2** (12 mg).

Fraction B4 (400 mg) was chromatographed on a Sephadex LH-20 column eluted with MeOH to obtain eight subfractions (B4.1–B4.8). Subfraction B4.2 (100 mg) was purified by RP-18 reversed-phase column chromatography using MeOH–H_2_O (1:1, *v*/*v*) to yield compound **4** (5 mg). Subfraction B4.3 (50 mg) was purified by silica gel column chromatography using CH_2_Cl_2_–MeOH (9:1, *v*/*v*) to obtain compound **8** (15 mg). Compound **6** (15 mg) was obtained from subfraction B4.6 (50 mg) after silica gel column chromatography using EtOAc–MeOH (9:1, *v*/*v*). Subfraction B4.7 (80 mg) was further purified on Sephadex LH-20 using MeOH to yield compound **3** (3 mg). To the best of our knowledge, these compounds have not previously been reported as isolated constituents of *A. sylvestris*.

*Compound* **1** *(3S,5R,6R,9S-tetrahydroxymegastigman-7-ene)*

White solid; molecular formula C_13_H_24_O_4_. ^1^H NMR (600 MHz, CD_3_OD): δ_H_ (ppm); 1.47 (1H, m, H-2a), 1.67 (1H, t, *J* = 12.0 Hz, H-2b), 4.08 (1H, m, H-3), 1.76 (2H, m, H-4), 6.09 (1H, dd, *J* = 1.2, 15.6 Hz, H-7), 5.81 (1H, dd, *J* = 6.0, 15.6 Hz, H-8), 4.36 (1H, m, H-9), 1.28 (3H, d, *J* = 6.6 Hz, Me-10), 0.89 (3H, s, Me-11), 1.24 (3H, s, Me-12), 1.12 (3H, s, Me-13). ^13^C NMR (150 MHz, CD_3_OD): δ_C_ 40.6 (C-1), 46.3 (C-2), 65.1 (C-3), 45.5 (C-4), 77.7 (C-5), 78.8 (C-6), 130.9 (C-7), 135.9 (C-8), 69.4 (C-9), 24.0 (C-10), 27.5 (C-11), 26.1 (C-12), and 27.1 (C-13).

*Compound* **2** *(Myricetin-3-O-rutinoside)*

Yellow solid; molecular formula C_27_H_30_O_17_. HR-ESI-MS: *m*/*z* 649 [M + Na]^+^. ^1^H NMR (600 MHz, CD_3_OD): δ_H_ (ppm); 6.19 (1H, d, *J* = 1.8 Hz, H-6), 6.37 (1H, d, J = 1.8 Hz, H-8), 7.32 (2H, s, H-2′, H-6′), 5.05 (1H, d, *J* = 7.8 Hz, H-1″), 3.54 (1H, dd, *J* = 7.8, 9.0 Hz, H-2″), 3.44 (1H, m, H-3″), 3.29 (2H, m, H-4″ and H-5″), 3.82 (1H, d, *J* = 11.2 Hz, H-6″a), 3.41 (1H, m, H-6″b), 4.54 (1H, br. s, H-1‴), 3.66 (1H, br. d, *J* = 3.6 Hz, H-2‴), 3.59 (1H, dd, *J* = 9.0, 3.6 Hz, H-3‴), 3.29 (1H, t, *J* = 9.0 Hz, H-4‴), 3.47 (1H, m, H-5‴), and 1.15 (3H, d, *J* = 6.0 Hz, Me-6‴). ^13^C NMR (150 MHz, CD_3_OD): δ_C_ (ppm); 158.6 (C-2), 135.7 (C-3), 179.4 (C-4), 163.0 (C-5), 99.9 (C-6), 166.0 (C-7), 94.8 (C-8), 158.5 (C-9), 105.6 (C-10), 122.1 (C-1′), 110.3 (C-2′), 146.3 (C-3′), 138.0 (C-4′), 146.4 (C-5′), 110.4 (C-6′), 104.8 (C-1″), 75.7 (C-2″), 78.2 (C-3″), 71.4 (C-4″), 77.3 (C-5″), 68.6 (C-6″), 102.4 (C-1‴), 72.1 (C-2‴), 72.2 (C-3‴), 74.0 (C-4‴), 69.7 (C-5‴), and 17.8 (C-6‴).

*Compound* **3** *(Phenyl ethyl-O-α-L-rhamnopyranosyl-(1→2)-β-D-glucopyranoside)*

White solid; molecular formula C_20_H_30_O_10_. ^1^H NMR (600 MHz, CD_3_OD): δ_H_ (ppm); 7.26–7.29 (4H, m, H-2, H-3, H-5, H-6), 7.19 (1H, m, H-4), 2.97 (2H, m, H-7), 3.76 (1H, m, H-8a), 4.10 (1H, m, H-8b), 4.39 (1H, d, *J* = 7.8 Hz, H-1′), 3.41 (1H, m, H-2′), 3.48 (1H, t, *J* = 9.0 Hz, H-3′), 3.30 (1H, t, *J* = 9.0 Hz, H-4′), 3.27 (1H, m, H-5′), 3.87 (1H, dd, *J* = 2.4, 12.0 Hz, H-6′a), 3.68 (1H, dd, *J* = 5.4, 12.0 Hz, H-6′b), 5.22 (1H, d, *J* = 1.2 Hz, H-1″), 3.95 (1H, dd, *J* = 1.8, 3.6 Hz, H-2″), 3.71 (1H, dd, *J* = 9.0, 3.6 Hz, H-3″), 3.40 (1H, t, *J* = 9.0 Hz, H-4″), 4.09 (1H, m, H-5″), and 1.25 (3H, d, *J* = 6.0 Hz, Me-6″). ^13^C-NMR (150 MHz, CD_3_OD): δ_C_ (ppm); 139.8 (C-1), 129.4 (C-2), 129.9 (C-3), 127.2 (C-4), 129.9 (C-5), 129.4 (C-6), 37.5 (C-7), 72.0 (C-8), 105.4 (C-1′), 79.2 (C-2′), 78.8 (C-3′), 70.3 (C-4′), 76.4 (C-5′), 61.3 (C-6′), 102.2 (C-1″), 72.4 (C-2″), 72.6 (C-3″), 74.2 (C-4″), 70.5 (C-5″), 18.2 (C-6″).

*Compound* **4** *(2-O-(trans-coumaroyl)malic acid 1-methyl ester)*

White solid. ^1^H NMR (CD_3_OD): δ_H_ (ppm); 6.25 (2H, d, *J* = 8.4 Hz, H-3′, H-5′), 7.48 (2H, d, *J* = 8.4 Hz, H-2′, H-6′), 7.64 (1H, d, *J* = 16.2 Hz, H-7′), 6.39 (1H, d, *J* = 16.2 Hz, H-8′), 3.71 (3H, s, OCH_3_), 5.38 (1H, dd, *J* = 3.6, 9.6 Hz, H-2), 2.98 (1H, dd, *J* = 3.6, 16.2 Hz, H-3a), and 2.85 (1H, dd, *J* = 9.6, 16.2 Hz, H-3b). ^13^C NMR (150 MHz, CD_3_OD): δ_C_ (ppm); 172.9 (C-1); 176.3 (C-4); 168.7 (C-9′); 72.8 (C-2); 38.2 (C-3); 52.3 (OCH_3_).

*Compound* **5** *(Adenosine)*

White solid; molecular formula C_10_H_13_N_5_O_4_. ^1^H NMR (600 MHz, CD_3_OD): δ_H_ (ppm); 8.20 (1H, s, H-2), 8.32 (1H, s, H-8), 5.99 (1H, d, *J* = 6.0 Hz, H-1′), 4.76 (1H, dd, *J* = 5.4, 6.0 Hz, H-2′), 4.35 (1H, dd, *J* = 5.4, 4.8 Hz, H-3′), 4.19 (1H, m, H-4′), 3.91 (1H, dd, *J* = 3.0, 12.6 Hz, H-5′a), and 3.77 (1H, dd, *J* = 3.0, 12.6 Hz, H-5′b); ^13^C-NMR (150 MHz, CD_3_OD): 152.4 (C-2), 149.0 (C-4), 119.3 (C-5), 156.2 (C-6), 139.9 (C-8), 87.9 (C-1′), 73.4 (C-2′), 70.6 (C-3′), 85.9 (C-4′), 61.6 (C-5′).

*Compound* **6** *(Gallic acid)*

White solid; molecular formula C_7_H_6_O_5_. ^1^H NMR (600 MHz, CD_3_OD): δ_H_ (ppm); 7.06 (2H, br. s, H-2 and H-6). ^13^C NMR (CD_3_OD, 150 MHz): δ_C_ (ppm); 127.7 (C-1), 110.1 (C-2 and C-6), 145.9 (C-3 and C-5), 137.6 (C-4), and 174.3 (COOH).

*Compound* **7** *(5-(8Z-heptadecenyl)resorcinol)*

White solid; molecular formula C_23_H_38_O_2_. ^1^H NMR (600 MHz, CD_3_OD): δ_H_ (ppm); 6.18 (1H, t, *J* = 2.1 Hz, H-2), 6.23 (2H, d, *J* = 2.1 Hz, H-4 and H-6), 2.47 (2H, t, *J* = 7.4 Hz, H-1′), 1.56 (2H, m, H-2′), 1.26–1.33 (8H, m, H-3′–H-6′), 2.02 (2H, m, H-7′), 5.35 (2H, m, H-8′ and H-9′), 2.02 (2H, m, H-10′), 1.26–1.33 (8H, m, H-11′–H-14′), 1.31 (2H, m, H-15′), 1.26 (2H, m, H-16′), and 0.87 (3H, t, J = 7.0 Hz, Me-17′). ^13^C-NMR (150 MHz, CDCl_3_): δ_C_ (ppm); 156.6 (C-1 and C-3), 100.2 (C-2), 108.0 (C-4 and C-6), 146.1 (C-5), 35.8 (C-1′), 31.0 (C-2′), 29.0–29.8 (C-3′–C-6′), 27.2 (C-7′), 129.9 (C-8′), 129.8 (C-9′), 27.3 (C-10′), 29.0–29.7 (C-11′–C-14′), 31.8 (C-15′), 22.6 (C-16′), 14.1 (C-17′).

*Compound* **8** *(Mandelic acid)*

White solid; molecular formula C_8_H_8_O_3_. ^1^H NMR (600 MHz, CD_3_OD): δ_H_ 7.36 (2H, m, H-2 and H-6), 7.49 (2H, m, H-3 and H-5), 7.32 (1H, m, H-4), and 5.02 (1H, s, H-7). ^13^C NMR (150 MHz, CD_3_OD): δ_C_ (ppm); 141.6 (C-1), 127.9 (C-2 and C-6), 129.3 (C-3 and C-5), 129.0 (C-4), 75.3 (C-7), and 178.5 (COOH).

### 2.4. α-Glucosidase Inhibitory Assay

The α-glucosidase inhibitory assay was performed as previously described with minor modifications [10]. α-glucosidase from *Saccharomyces cerevisiae* was obtained from Sigma-Aldrich, St. Louis, MO, USA; catalogue number G5003. The isolated compounds were dissolved in DMSO (20 mg/mL) and diluted to final concentrations of 256, 64, 16, and 4 µg/mL. The final concentration of DMSO in the reaction mixture was 0.64% (*v*/*v*). A corresponding vehicle control containing the same concentration of DMSO but without the tested compound was included. At this concentration (<1%, *v*/*v*), DMSO was considered not to interfere with the α-glucosidase assay. Acarbose was used as the positive control. Samples were incubated with α-glucosidase (0.4 U/mL) in 100 mM PBS (pH 6.8) at 37 °C for 10 min, followed by the addition of p-nitrophenyl-α-D-glucopyranoside (pNPG, 2.5 mM). After incubation at 37 °C for 30 min, the reaction was terminated with 0.2 M Na_2_CO_3_, and absorbance was measured at 410 nm. The percentage inhibition was calculated as: Inhibition (%) = [1 − (A_s_ − A_sb_)/(A_c_ − A_cb_)] × 100, where A_s_ and A_sb_ represent the absorbance of the test sample with and without enzyme, respectively, and A_c_ and A_cb_ represent the corresponding control absorbances in the absence of inhibitor. All measurements were performed in technical triplicate using three parallel wells. IC_50_ values were estimated from concentration–response curves by nonlinear regression using a four-parameter logistic model in GraphPad Prism version 10.0.

### 2.5. AChE Inhibitory Assay

The acetylcholinesterase (AChE) inhibitory activity was determined according to the method of Ingkaninan et al. with slight modifications [14,15]. Acetylcholinesterase from *Electrophorus electricus* (electric eel), acetylthiocholine iodide (ATCI), and 5,5′-dithiobis-(2-nitrobenzoic acid) (DTNB) were obtained from Sigma-Aldrich (St. Louis, MO, USA). The isolated compounds were dissolved in DMSO (20 mg/mL) and tested at final concentrations of 2–512 µg/mL. The final concentration of DMSO in the reaction mixture was 0.64% (*v*/*v*). Donepezil was used as the positive control. In this assay, 100 µL of the test solution was mixed with 80 µL of 50 mM Tris–HCl buffer (pH 8.0) and 10 µL of AChE solution (0.25 IU/mL) in a 96-well plate, followed by pre-incubation at 25 °C for 15 min. Subsequently, 5 µL of 5,5′-dithiobis-(2-nitrobenzoic acid) (DTNB, 2.4 mM) and 5 µL of acetylthiocholine iodide (ATCI, 70 mM) were added to initiate the reaction, followed by incubation at 25 °C for another 15 min. The absorbance was measured at 412 nm using a microplate reader. Tris–HCl buffer was used as the negative control. In addition, a vehicle control containing 0.64% (*v*/*v*) DMSO without the tested compound was included to account for any solvent effect. At this concentration (<1%, *v*/*v*), DMSO was considered not to interfere with AChE activity under the assay conditions. The percentage inhibition of AChE activity was calculated as: Inhibition (%) = [(A_c_ − A_s_)/A_c_] × 100% where A_c_ and A_s_ represent the absorbance of the negative control (or blank sample) and the tested sample, respectively. All measurements were performed in technical triplicate using three parallel wells, and IC_50_ values were obtained from concentration–response curves.

### 2.6. DPPH Radical-Scavenging Assay

The DPPH assay was selected as a simple chemical method to assess the free-radical-scavenging capacity of the isolated compounds. Because this assay reflects hydrogen/electron donation in a cell-free system, the results were interpreted specifically as 2,2-diphenyl-1-picrylhydrazyl (DPPH) radical-scavenging activity rather than as comprehensive biological antioxidant activity [10,16]. The final concentration of DMSO in the reaction mixture was 0.64% (*v*/*v*). A corresponding vehicle control containing the same concentration of DMSO but without the tested compound was included. At this concentration (<1%, *v*/*v*), DMSO was considered not to interfere with the DPPH radical-scavenging assay. Quercetin was used as the positive control. In each well of a 96-well microplate, 100 µL of the sample solution was mixed with 100 µL of freshly prepared DPPH solution (0.2 mM in methanol). The reaction mixtures were incubated in the dark at room temperature for 30 min, after which the absorbance was measured at 517 nm using a microplate reader. The radical-scavenging activity was calculated according to the following equation: Scavenging activity (%) = [(A_c_ − A_s_)/A_c_]*100 in which A_c_ and A_s_ are the absorbance values of the control and the test sample, respectively. All measurements were performed in technical triplicate using three parallel wells, and IC_50_ values were calculated from concentration–response curves.

### 2.7. Antimicrobial Activity Tests

The antimicrobial activities of the isolated compounds were evaluated against representative Gram-positive bacteria (*Staphylococcus aureus* ATCC 13709 and *Bacillus subtilis* ATCC 6633), Gram-negative bacteria (*Escherichia coli* ATCC 25922 and *Pseudomonas aeruginosa* ATCC 15442), and the yeast *Candida albicans* ATCC 10231 using the broth microdilution method according to the Clinical and Laboratory Standards Institute (CLSI) guidelines with slight modifications [17,18]. The reference strains were obtained from the American Type Culture Collection (ATCC) and maintained at −80 °C until use. Mueller–Hinton broth (MHB) and Tryptic Soy Broth (TSB) were used as culture media. The compounds were dissolved in DMSO and serially diluted in MHB to 0.5–256 μg/mL, with a final DMSO concentration ≤1% (*v*/*v*). Ampicillin and cefotaxime served as antibacterial positive controls and nystatin as the antifungal control. Microbial suspensions were adjusted to approximately 5 × 10^5^ CFU/mL, and 190 μL was mixed with 10 μL of each test solution in sterile 96-well plates and incubated at 37 °C for 18–24 h. Growth and sterility controls contained inoculated medium without test compounds and culture medium alone, respectively. Microbial growth was assessed at 590 nm, and the MIC was defined as the lowest concentration completely inhibiting visible growth. All antimicrobial assays were performed in three independent experiments with triplicate measurements.

### 2.8. Substrate-Similarity-Based Prioritization of S. aureus Targets

A customized semiquantitative substrate-similarity framework was proposed to prioritize potential *S. aureus* targets for compound **7** (Figure 1).

A total of 53 unique proteins were collected from the literature-curated target set reported by Cherkasov et al. after removal of duplicate entries and classified according to their biological functions [19]. For each target, the query compound was compared with its principal physiological substrate or, for macromolecular substrates (e.g., acyl-ACP, peptides, or protein complexes), with the biologically relevant substrate fragment expected to occupy the ligand-binding pocket.

The substrate-likeness target score (SLTS) was calculated by integrating six complementary similarity descriptors, including two-dimensional structural similarity, biologically relevant substrate-fragment similarity, three-dimensional shape similarity, pharmacophore similarity, physicochemical-property similarity, and lipophilic-chain similarity:

*SLTS_(t,s)_* = 100 (0.15*S_2D_* + 0.2*S_fragment_* + 0.2*S_shape_* + 0.15*S_pharmacophore_* + 0.1*S_physchem_* + 0.2*S_chain_*) (1)

where *S_2D_*, *S_fragment_*, *S_shape_*, *S_pharmacophore_*, *S_physchem_*, and *S_chain_* represent two-dimensional structural similarity, biologically relevant substrate-fragment similarity, three-dimensional shape similarity, pharmacophore similarity, physicochemical-property similarity, and lipophilic-chain similarity, respectively. Detailed scoring information and target data are provided in Supplementary Tables S1–S4. Greater weights were assigned to substrate-fragment, three-dimensional shape, and lipophilic-chain similarities because these descriptors more directly capture substrate-recognition features within ligand-binding pockets, particularly for enzymes acting on carrier-bound or lipid-associated substrates, for which whole-molecule structural similarity alone may underestimate biological relevance. Individual descriptor calculations are summarized in Figure 1.

For proteins possessing multiple physiological substrates, a target-level score was calculated by combining the highest substrate score with the weighted average of all relevant substrate scores:

*SLTS_target_* = 0.80 × *maximum* (*rs* × *SLTS*_(*t*,*s*)_) + 0.20 × [Σ(*rs* × *SLTS_(t_*_,*s)*_)/Σ*rs*] (2)

where *rs* represents the biological relevance assigned to each substrate. Principal and target-specific substrates were given higher weights than common cofactors such as ATP, GTP, NAD(P)H, and FAD.

Because substrate similarity is inherently more informative for enzymes with defined small-molecule or lipid substrates than for toxins, regulatory proteins, or large macromolecular complexes, a substrate-applicability coefficient (*A_t_*) was introduced (Table S4). The final priority score was calculated as:

*Pt* = *SLTS_target_* × [0.75 + 0.25(*A_t_*)] (3)

All the proteins were ranked according to their final priority scores. Targets were selected for subsequent molecular docking based on their ranking, biological relevance to *S. aureus*, availability of a defined ligand-binding pocket, and availability of a reliable three-dimensional structure.

### 2.9. Molecular Docking Study

Molecular docking was performed to provide mechanistic interpretation of the experimentally observed activities using Molecular Operating Environment (MOE, version 2015.10, Chemical Computing Group, Montreal, Canada) [20]. Human intestinal maltase–glucoamylase (α-glucosidase, PDB ID: 2QMJ) [21] was selected as a physiologically relevant intestinal α-glucosidase model, whereas human acetylcholinesterase (AChE, PDB ID: 4EY7) [22] directly corresponds to the AChE inhibitory assay. For the antibacterial activity, candidate S. aureus targets were prioritized using the substrate-similarity framework described in Section 2.9. Protein crystal structures were retrieved from the Protein Data Bank and prepared by removing co-crystallized ligands, non-essential heteroatoms, and water molecules, followed by protonation using the Protonate3D module and energy minimization with the Amber10:EHT force field [23]. Protein structures were inspected for missing residues and incomplete side chains before docking. The docking protocol was validated by redocking the co-crystallized ligand into the corresponding binding site of each protein. The protocol was considered reliable when the predicted binding pose closely reproduced the experimental conformation with a root mean square deviation (RMSD) < 2.0 Å while maintaining the key protein–ligand interactions observed in the crystal structure [24]. Compounds **1** and **7** were docked into α-glucosidase, whereas only compound **7** was docked into AChE and antibacterial targets following the reported procedures [10,25,26]. The binding site was defined based on the co-crystallized ligand. Docking calculations were performed using the Triangle Matcher placement method followed by Induced Fit refinement [27]. The best docking pose was selected according to the London dG and GBVI/WSA dG scoring functions. Protein–ligand interactions were visualized using ChimeraX 1.10 and Discovery Studio Visualizer 2024.

### 2.10. Molecular Dynamics

Molecular dynamics (MD) simulations were performed using GROMACS 2024.1 with the CHARMM36m force field to evaluate the stability of the docked protein–ligand complexes [28]. Ligand parameters were generated using the CHARMM General Force Field (CGenFF). The α-glucosidase complexes with compounds **1** and **7**, together with the AChE–compound **7**, were solvated in a TIP3P water box and neutralized with Na^+^ or Cl^−^ ions [10]. The resulting simulation systems contained 209,605 atoms, 209,627 atoms, and 160,936 atoms, respectively. After energy minimization, the systems were equilibrated under NVT ensemble for 1ns and NPT ensembles for 2ns before a 100 ns production run at 300 K and 1 bar under periodic boundary conditions. Trajectory coordinates were saved every 10 ps for subsequent analyses. Long-range electrostatic interactions were treated using the Particle Mesh Ewald (PME) method, and hydrogen-containing bonds were constrained using the LINCS algorithm. Trajectory analyses were performed using the built-in GROMACS utilities. Structural stability and conformational changes were evaluated by calculating the RMSD, root mean square fluctuation (RMSF), radius of gyration (Rg), and free-energy landscape (FEL). MM/PBSA binding free-energy calculations were performed using snapshots extracted from the equilibrated portion of the MD trajectories. The binding free energy was estimated from molecular-mechanics and solvation-energy contributions, including van der Waals, electrostatic, polar solvation, and nonpolar solvation terms.

### 2.11. In Silico Drug-likeness and ADME Prediction

The drug-likeness and pharmacokinetic properties of compounds **1** and **7** were predicted using the SwissADME platform. The analysis included molecular weight, lipophilicity, topological polar surface area, hydrogen-bond donors and acceptors, gastrointestinal absorption, blood–brain barrier permeation, P-glycoprotein substrate status, and compliance with Lipinski and Veber criteria [29,30]. The predicted properties were interpreted according to established SwissADME-based approaches [31].

### 2.12. Statistical Analysis

Quantitative data are presented as mean ± standard deviation (SD). IC_50_ values were obtained from concentration–response curves as described in the corresponding assay sections. No formal inferential statistical comparisons or post hoc analyses were performed, as the study was designed primarily to estimate the biological activity parameters of individual isolated compounds.

## 3. Results

### 3.1. Isolation and Structural Identification of Compounds from Ardisia sylvestris

Repeated chromatographic separation of the EtOH extract of *A. sylvestris* leaves, including silica gel, Sephadex LH-20, Diaion HP-20, and reversed-phase RP-18 column chromatography, afforded eight pure compounds (Figure 2). Their structures were identified based primarily on 1D and 2D NMR analyses (^1^H, ^13^C, COSY, HSQC, and HMBC), together with comparison with reported spectroscopic data. MS data were additionally available for compound **2**. The isolated compounds were identified as 3*S*,5*R*,6*R*,9*S*-tetrahydroxymegastigman-7-ene (**1**), myricetin-3-*O*-rutinoside (**2**), phenyl ethyl-*O*-α-L-rhamnopyranosyl-(1→2)-β-D-glucopyranoside (**3**), 2-*O*-(trans-coumaroyl)malic acid 1-methyl ester (**4**), adenosine (**5**), gallic acid (**6**), 5-(8*Z*-heptadecenyl)resorcinol (**7**), and mandelic acid (**8**) (Figure 3).

Compound **1** was obtained as a white amorphous solid. The ^1^H NMR spectrum immediately suggested a megastigmane skeleton through the presence of three tertiary methyl singlets at δ_H_ 0.89 (Me-11), 1.12 (Me-13), and 1.24 (Me-12), together with a secondary methyl doublet at δ_H_ 1.28 (*J* = 6.6 Hz, Me-10). Two oxygenated methine signals at δ_H_ 4.08 (H-3) and 4.36 (H-9), as well as a pair of trans-olefinic protons at δ_H_ 6.09 (*J* = 1.2, 15.6 Hz, H-7) and 5.81 (*J* = 6.0, 15.6 Hz, H-8), indicated a hydroxylated megastigmane bearing an E-configured double bond. The ^13^C NMR spectrum displayed 13 carbon resonances comprising four methyl carbons, two methylene carbons, two oxygenated methines, two olefinic methines, and three quaternary carbons. The COSY spectrum established two principal proton spin systems (H-2/H-3/H-4 and H-7/H-8/H-9/Me-10), whereas the HMBC correlations from H-7 to C-6/C-9, H-8 to C-6/C-9/C-10, and Me-10 to C-8/C-9 unambiguously established the Δ^7^ double bond and the position of the C-10 methyl substituent. The remaining structural assignments were completed using HSQC and HMBC data, and the spectroscopic features were fully consistent with those reported for 3*S*,5*R*,6*R*,9*S*-tetrahydroxymegastigman-7-ene [32].

Compound **2** was isolated as a yellow amorphous powder. Its HR-ESI-MS spectrum exhibited a sodium adduction at *m*/*z* 649 [M + Na]^+^, consistent with the molecular formula C_27_H_30_O_17_. The aromatic proton pattern in the ^1^H NMR spectrum, consisting of two meta-coupled signals at δ_H_ 6.19 (H-6) and 6.37 (H-8) together with a two-proton singlet at δ_H_ 7.32 (H-2′/H-6′), was characteristic of a myricetin nucleus. Two anomeric proton signals at δ_H 5.05 (*J* = 7.8 Hz, H-1″) and 4.54 (br.s, H-1‴) indicated the presence of two sugar residues. The large coupling constant of H-1″ established a β-D-glucopyranosyl unit, whereas the broad singlet of H-1‴ and the methyl doublet at δ_H_ 1.15 (*J* = 6.0 Hz, Me-6‴) supported an α-L-rhamnopyranosyl residue. Complete assignment of the proton and carbon resonances was achieved by HSQC analysis, while the HMBC cross-peak from H-1″ to C-3 of the flavonol aglycone demonstrated glycosylation at C-3. In addition, the correlation between H-1‴ and C-6″ established the rutinosyl linkage. These data were in agreement with those reported for myricetin-3-*O*-rutinoside [33].

Compound **3** was obtained as a white solid. The ^1^H NMR spectrum displayed characteristic signals attributable to a phenylethanol moiety together with two anomeric proton signals at δ_H_ 4.39 (*J* = 7.8 Hz, H-1′) and 5.22 (*J* = 1.2 Hz, H-1″), indicating the presence of β-D-glucopyranose and α-L-rhamnopyranose residues. The methyl doublet at δ_H_ 1.25 (*J* = 6.0 Hz) further confirmed the rhamnose unit. Analysis of the COSY spectrum established three major proton spin systems corresponding to the aglycone and the two sugar moieties. The HMBC correlation from H-1′ to C-8 established the attachment of glucose to the phenylethanol skeleton, whereas the correlation from H-1″ to C-2′ confirmed the α-L-rhamnopyranosyl-(1→2)-β-D-glucopyranosyl linkage. Comparison with reported spectroscopic data identified compound **3** as phenyl ethyl-O-α-L-rhamnopyranosyl-(1→2)-β-D-glucopyranoside [34].

Compound **4** was isolated as a white solid. Its ^1^H NMR spectrum exhibited a typical AA′BB′ aromatic proton system together with two trans-coupled olefinic protons (*J* = 16.2 Hz), indicating the presence of a trans-*p*-coumaroyl moiety. Signals corresponding to one oxygenated methine, one methylene group, and one methoxy group suggested a substituted malic acid methyl ester skeleton. The structure was established by HSQC and HMBC analyses, particularly the correlation from the methoxy protons to the ester carbonyl carbon, and was identified as 2-*O*-(trans-coumaroyl)malic acid 1-methyl ester [35].

Compound **5** was obtained as a white solid. The characteristic singlets at δ_H_ 8.20 (H-2) and 8.32 (H-8) in the ^1^H NMR spectrum, together with resonances attributable to a ribofuranosyl moiety, suggested an adenosine skeleton [36]. The assignments were confirmed by HSQC analysis and comparison of the ^1^H and ^13^C NMR data with published values [37], leading to the identification of compound **5** as adenosine.

The remaining compounds were readily identified by comparison of their spectroscopic data with authentic literature reports. Compound **6** was assigned as gallic acid based on its characteristic symmetrical aromatic proton pattern and highly oxygenated benzoic acid skeleton [38]. Compound **7** was identified as 5-(8*Z*-heptadecenyl)resorcinol from the typical 1,3,5-trisubstituted resorcinol aromatic system together with an unsaturated C_17_ alkyl side chain containing a cis double bond [39]. Compound **8** was characterized as mandelic acid by the presence of a monosubstituted benzene ring, an oxygenated methine carbon, and a carboxylic acid functionality. The spectroscopic data of compounds **6**–**8** showed excellent agreement with those previously reported.

### 3.2. In Vitro Biological Activities and Molecular Simulation Insights

The isolated compounds were evaluated for α-glucosidase inhibition, AChE inhibition, and antioxidant activity using the DPPH radical-scavenging assay. The results are summarized in Table 1.

Marked differences in biological activities were observed among the isolated constituents. In the α-glucosidase inhibition assay, compound **7** exhibited the highest inhibitory activity with an IC_50_ value of 7.56 ± 0.34 μg/mL, representing approximately a 20-fold higher potency than the positive control acarbose (IC_50_ = 151.09 ± 6.22 μg/mL). Compound **1** also showed pronounced inhibitory activity (IC_50_ = 16.79 ± 0.84 μg/mL), followed by compound **3** (IC_50_ = 52.00 ± 1.25 μg/mL). Compound **4** displayed moderate inhibition (IC_50_ = 125.09 ± 2.74 μg/mL), whereas compound **6** showed relatively weak activity (IC_50_ = 230.40 ± 5.58 μg/mL). In contrast, compounds **2**, **5**, and **8** were inactive under the experimental conditions (IC_50_ > 256 μg/mL).

The AChE inhibitory assay revealed a more selective activity profile. Among all isolated compounds, only compound **7** exhibited measurable inhibition, with an IC_50_ value of 14.75 ± 1.07 μg/mL, whereas compounds **1**–**6** and **8** showed no detectable activity at the highest tested concentration (IC_50_ > 256 μg/mL). As expected, the reference inhibitor donepezil showed potent activity with an IC_50_ value of 0.028 ± 0.002 μg/mL.

In the DPPH radical-scavenging assay, compound **7** again displayed the strongest antioxidant activity (IC_50_ = 10.04 ± 1.05 μg/mL), which was comparable to that of quercetin (IC_50_ = 8.65 ± 0.68 μg/mL). Compounds **1** and **6** also demonstrated considerable free radical-scavenging activities with IC_50_ values of 36.01 ± 2.19 and 35.19 ± 1.50 μg/mL, respectively. Moderate antioxidant activities were observed for compounds **5**, **8**, and **4**, whereas compounds **2** and **3** showed negligible radical-scavenging effects within the tested concentration range.

To further illustrate the concentration-dependent activity of the most active constituent, representative concentration–response curves for compound **7** and the corresponding reference controls are shown in Figure 4.

Overall, compound **7** showed the most favorable biological profile, with pronounced α-glucosidase inhibition, measurable AChE inhibition, and strong DPPH radical-scavenging activity.

To gain mechanistic insights into the in vitro activities, molecular docking was performed using human α-glucosidase (PDB ID: 2QMJ) [21] for compounds **1** and **7**, and AChE (PDB ID: 4EY7) for compound **7** [22]. The docking protocol was first validated by redocking the co-crystallized ligands into their respective binding sites. As shown in Figure 5, the redocked poses closely overlapped with the crystallographic conformations, with RMSD values of 1.8893 Å for α-glucosidase and 0.9825 Å for AChE. The corresponding London dG/GBVI-WSA dG scores were −11.6/−9.9 kcal/mol for acarbose and −12.3/−9.2 kcal/mol for donepezil. The redocked poses also reproduced the major interactions with residues within the respective binding sites, including Asp203, Asp327, Asp443, Asp542, Arg526, Tyr299, Phe575, and His600 for α-glucosidase and Trp86, Trp286, Phe295, Tyr337, Phe338, and Tyr341 for AChE [21,22].

Compounds **1** and **7** were docked into the active site of α-glucosidase following the validated protocol. As shown in Figure 6A,B, both compounds occupied the same substrate-binding pocket as the co-crystallized ligand acarbose [10], with partial overlap of their binding orientations. Compound **1** adopted a binding orientation stabilized by conventional hydrogen bonds with Asp327 and Asp542, together with π-anion and hydrophobic interactions involving Tyr299, Phe575, Met444, and Trp406 (Figure 6A). Compound **7** exhibited a similar binding orientation but formed a more favorable interaction network, including hydrogen bonds with Asp327 and Asp443, while its long hydrophobic side chain occupied the hydrophobic channel lined by Trp406, Tyr605, Ala576, and neighboring residues (Figure 6B). Consistently, compound **7** showed more favorable docking scores (London dG = −10.48 kcal/mol; GBVI/WSA dG = −7.59 kcal/mol) than compound **1** (London dG = −9.58 kcal/mol; GBVI/WSA dG = −6.32 kcal/mol). The docking scores and major protein–ligand interactions of the selected compounds and reference ligands are summarized in Table 2.

To further evaluate the stability of the docked complexes, 100 ns molecular dynamics simulations were performed. As shown in Figure 6C, the apo enzyme remained relatively stable throughout the simulation, with RMSD values fluctuating around 1 Å. The α-glucosidase–compound **7** complex rapidly reached equilibrium and maintained RMSD values of approximately 1.8–2.0 Å during the production phase. In contrast, the α-glucosidase–compound **1** complex exhibited a gradual increase in RMSD after approximately 45 ns, fluctuating around 2.5–3.0 Å, indicating greater structural flexibility [28]. RMSF showed similar flexibility profiles for both complexes, with only minor fluctuations observed in several loop regions and no marked increase in mobility around the catalytic residues (Figure 6D) [10].

Furthermore, the FEL showed that the α-glucosidase–compound **7** complex occupied a more concentrated low-energy basin than the α-glucosidase–compound **1** complex, suggesting a more stable conformational state during the simulation (Figure 6E,F). The MM/PBSA binding free-energy calculations further supported the molecular docking and MD results. Compound **7** exhibited a more favorable binding free energy (ΔG_bind_ = −116.4 ± 7.9 kJ/mol) than compound **1** (ΔG_bind_ = −88.3 ± 8.7 kJ/mol), indicating a stronger binding affinity toward α-glucosidase. For α-glucosidase, compound **7** showed a markedly stronger van der Waals contribution than compound **1**, which accounted for most of the difference in the calculated binding free energies (Table S5). Overall, the docking and MD results showed a more favorable binding profile for compound **7** than for compound **1**, consistent with their relative α-glucosidase inhibitory activities.

On the other hand, compound **7** was docked into the active-site gorge of human AChE. The results are visualized in Figure 6.

As shown in Figure 7A, compound **7** occupied a binding orientation similar to that of the co-crystallized ligand donepezil [40], with partial overlap of the two conformations. The ligand established hydrogen-bonding, π–π stacking, and hydrophobic interactions with key residues distributed along the catalytic gorge, including Trp86, Tyr337, Phe338, Trp286, Val294, Tyr341, Gly120, Glu202, and Gly448, spanning both the catalytic anionic site (CAS) and peripheral anionic site (PAS) [15]. Compound **7** yielded London dG and GBVI/WSA dG scores of −10.19 and −8.13 kcal/mol, respectively.

The stability of the docked complex was further evaluated by a 100 ns MD simulation. As shown in Figure 7B, the AChE–compound **7** complex rapidly reached equilibrium and remained stable throughout the simulation with RMSD values below approximately 1.2 Å. RMSF analysis showed low residue fluctuations across the catalytic gorge, including the CAS, PAS, oxyanion hole, and acyl-binding pocket (Figure 6C). The free-energy landscape revealed a well-defined low-energy basin, indicating a stable binding conformation during the simulation (Figure 6D). Consistently, MM/PBSA analysis predicted a favorable binding free energy (ΔG_bind_ = −105 ± 9 kJ/mol). The MM/PBSA energy decomposition (Table S5) showed that both van der Waals and electrostatic interactions contributed favorably to binding, partially offset by the unfavorable polar solvation energy.

In sum, the favorable docking, MD, and MM/PBSA profiles of compound **7** are consistent with its in vitro activities and provide plausible structural explanations for its interactions with the selected protein targets; however, direct biochemical and kinetic studies are still required to confirm the proposed mechanisms.

### 3.3. Antimicrobial Activity, Target Identification and Molecular Docking

#### 3.3.1. Antimicrobial Activity

The antimicrobial activities of compounds **1**–**8** were evaluated against representative Gram-positive bacteria (*S. aureus* ATCC 13709 and *B. subtilis* ATCC 6633), Gram-negative bacteria (*E. coli* ATCC 25922 and *P. aeruginosa* ATCC 15442), and the yeast *C. albicans* using the broth microdilution method [26]. The MIC values are summarized in Table 3.

Among the isolated compounds, only compound **7** showed measurable antibacterial activity, with an MIC of 64 μg/mL against *S. aureus*. No activity was observed against *B. subtilis*, *E. coli*, *P. aeruginosa*, or *C. albicans* at the highest tested concentration (MIC >256 μg/mL). Compounds **1**–**6** and **8** were inactive against all tested microorganisms (MIC >256 μg/mL). The reference agents showed MIC values of 0.125 and 32 μg/mL for ampicillin against *S. aureus* and *B. subtilis*, respectively; 0.5 and 8 μg/mL for cefotaxime against *B. subtilis* and *E. coli*, respectively; and 8 μg/mL for nystatin against *C. albicans*.

#### 3.3.2. Identification of Potential *S. aureus* Targets

To identify potential antibacterial targets of compound **7**, substrate-similarity analysis was performed against 53 unique *S. aureus* proteins derived from the literature-curated target set of Cherkasov et al. [19]. The proteins were ranked according to their similarity to the physiological substrates or biologically relevant substrate fragments of the corresponding targets (Table 4).

Enoyl-acyl carrier protein reductase (FabI) was ranked first, with an SLTS of 74.95 and a final priority score of 70.27. This high ranking was mainly attributable to its strong substrate-fragment, three-dimensional shape, and lipophilic-chain similarities, with corresponding values of 0.81, 0.82, and 0.92, respectively. These findings indicate that the C17:1 side chain of 5-(8Z-heptadecenyl)resorcinol resembles the hydrophobic enoyl-acyl portion of the FabI substrate. FabI catalyzes the NAD(P)H-dependent reduction of trans-2-enoyl-ACP during bacterial fatty-acid biosynthesis, with the acyl moiety accommodated within a predominantly hydrophobic substrate-binding cavity [41].

The 3-oxoacyl-acyl carrier protein synthase target class ranked second, with an SLTS of 69.25 and a final priority score of 64.92. This target class also showed high substrate-fragment, shape, and lipophilic-chain similarities of 0.74, 0.77, and 0.88, respectively. In *S. aureus*, this enzyme class includes FabF and FabH, which catalyze key condensation reactions during bacterial fatty-acid biosynthesis [42]. Although FabF and FabH act on distinct acyl substrates and participate in different stages of the pathway, both possess hydrophobic acyl-binding channels that may accommodate the long alkenyl chain of compound **7**.

A substantial decrease in score was observed for the third-ranked target, tyrosyl-tRNA synthetase, which had a final priority score of 29.90. Although the resorcinol moiety showed partial similarity to the aromatic portion of L-tyrosine, compound **7** lacked the amino acid and aminoacyl-adenylate features required for close substrate mimicry. Tryptophanyl-tRNA synthetase and signal peptidase Ib were ranked fourth and fifth, with final priority scores of 21.35 and 20.90, respectively.

Overall, the ranking strongly favored enzymes involved in bacterial fatty-acid biosynthesis. FabI was identified as the highest-priority individual target, whereas the high score obtained for the 3-oxoacyl-ACP synthase class supported further evaluation of both FabF and FabH [43]. Accordingly, FabI, FabF, and FabH were selected as the most promising targets for subsequent molecular docking analyses.

#### 3.3.3. Molecular Docking with FabI, FabF, and FabH

To further evaluate the highest-ranked antibacterial targets, compound **7** was docked into *S. aureus* FabI (PDB ID: 4FS3) [44] and structurally characterized FabF and FabH models (PDB IDs: 2GFX and 5BNR, respectively) [45,46]. FabI catalyzes the NADPH-dependent reduction of enoyl-ACP, whereas FabH initiates and FabF extends fatty-acid chains during bacterial type II fatty-acid biosynthesis [43,44,45,46]. The docking protocols were validated by redocking the co-crystallized inhibitors, yielding RMSD values below 1 Å. The London dG/GBVI/WSA dG scores of the redocked ligands were −12.19/−9.77, −10.47/−8.86, and −13.28/−10.38 kcal/mol for FabI, FabF, and FabH, respectively.

Compound **7** fitted into the hydrophobic ligand channels of all three enzymes (Figure 8). In FabI, its resorcinol moiety was positioned near Tyr157 and the NADP cofactor, while the long alkenyl chain formed extensive hydrophobic contacts with Phe96, Ala97, Met99, Leu102, Val201, Phe204, and Ile207. This binding mode is compatible with occupation of the enoyl-substrate channel adjacent to the redox cofactor [44]. FabI is an established essential target in *S. aureus*, and inhibitors may bind to the enzyme–NADP complex and block the terminal reduction step of fatty-acid elongation [44].

In FabF and FabH, compound **7** mainly occupied the elongated hydrophobic substrate channels through alkyl and π-related contacts. The FabF pose involved Arg206, Ser271, Pro272, Pro308, and Asn404, whereas the FabH pose was stabilized by residues including Trp32, Ile156, Leu189, Met207, Val212, Phe213, and Phe304. FabF catalyzes subsequent chain-elongation condensations, whereas FabH controls the initiating condensation and substrate selection [43].

Compound **7** gave London dG/GBVI/WSA dG scores of −10.51/−8.35 kcal/mol for FabI, −10.74/−7.46 kcal/mol for FabF, and −10.19/−8.13 kcal/mol for FabH (Table 5). Although the London dG values were similar, GBVI/WSA rescoring favored FabI, followed by FabH and FabF. Together with the target-prioritization results and the NADP-adjacent pose, these findings identify FabI as the most plausible primary target, while FabH may represent a secondary fatty-acid-biosynthesis target. This proposed mechanism is consistent with the moderate activity of compound **7** against *S. aureus* (MIC = 64 μg/mL), although direct enzyme-inhibition studies are required for confirmation.

### 3.4. In Silico Drug-likeness Assessment

SwissADME analysis revealed distinct physicochemical and pharmacokinetic profiles for compounds **1** and **7** (Figure 9; Table 6). Compound **1** showed a physicochemical profile largely within the optimal oral bioavailability space, whereas compound **7** deviated mainly in lipophilicity, insolubility, and flexibility (Figure 9).

Compound **1** was predicted to have high gastrointestinal absorption and complied with both Lipinski and Veber criteria [29,30], while compound **7** showed low gastrointestinal absorption, one Lipinski violation, and failed the Veber criterion. Neither compound was predicted to permeate the blood–brain barrier, suggesting limited passive central nervous system (CNS) exposure under the SwissADME model [31]. Compound **1** was predicted to be a P-glycoprotein substrate, whereas compound **7** was not. Compound **1** was not predicted to inhibit the major CYP isoforms evaluated, while compound **7** was predicted to inhibit CYP1A2, CYP2C19, and CYP2D6, indicating a higher potential for metabolism-related drug–drug interactions. No PAINS alerts were identified for either compound [47]. Overall, compound **1** showed a more favorable predicted drug-likeness profile, whereas compound **7** exhibited notable physicochemical and metabolic liabilities that may limit its oral developability.

## 4. Discussion

Previous investigations of *A. sylvestris* have mainly focused on preliminary phytochemical screening and the biological activities of crude extracts [4,5,6]. The leaves were reported to contain flavonoids, tannins, coumarins and other phenolic constituents, while ethyl acetate and ethanol extracts showed antioxidant and antibacterial effects of varying magnitude [1,2]. The present isolation of eight compounds belonging to several structural classes therefore provides a more specific chemical basis for some of these previously observed activities. Nevertheless, all isolated metabolites are known natural products, and their significance in the present study lies primarily in defining the constituent profile of *A. sylvestris* leaves and relating individual compounds to the measured biological effects rather than in chemical novelty.

The biological screening indicated that the activities were unevenly distributed among the isolated compounds. Compound **7**, identified as 5-(8Z-heptadecenyl)resorcinol, was consistently the most active constituent, whereas most of the remaining compounds were inactive or showed only weak-to-moderate effects. This profile is compatible with the amphiphilic character of alkylresorcinols, which combine a polar dihydroxybenzene moiety with a long lipophilic side chain [48]. Alkylresorcinols are known to display several biological properties, including radical-scavenging and antimicrobial effects, although their activity varies considerably with chain length, unsaturation, assay system and biological target [49]. Thus, the relatively broad profile of compound **7** is not unexpected, but the present data help clarify its contribution within the chemical mixture of *A. sylvestris* leaves.

The most pronounced result was obtained in the α-glucosidase assay. Compound **7** inhibited the enzyme with an IC_50_ value of 7.56 ± 0.34 μg/mL, compared with 151.09 ± 6.22 μg/mL for acarbose under the same experimental conditions. Although this difference corresponds to an approximately 20-fold lower mass concentration, it should be interpreted cautiously because mass-based comparisons do not account for molecular-weight differences, and inhibition values can vary substantially according to enzyme source, substrate and assay protocol. Nevertheless, compound **7** was also clearly more active than the other isolated metabolites, including compound **1** and compound **3**, which gave IC_50_ values of 16.79 ± 0.84 and 52.00 ± 1.25 μg/mL, respectively. The activity pattern therefore suggests that the resorcinol ring and the long alkenyl chain together provide a more favorable combination for α-glucosidase binding than the more compact megastigmane structure of compound **1** or the highly polar glycosidic structure of compound **3**.

Beyond direct α-glucosidase inhibition, plant-derived natural products may also exert antidiabetic effects through downstream mechanisms. For example, cyanoglycosides from Moringa oleifera have been reported to alleviate diabetic nephropathy through modulation of the PFKFB3/TGF-β1/Smads pathway [50], suggesting that future studies of compound **7** should extend to cellular and in vivo models of glucose dysregulation and diabetes-related complications.

The computational results provide a reasonable structural explanation for this experimental ranking. Both compounds **1** and **7** were accommodated in the substrate-binding region of α-glucosidase, but compound **7** obtained more favorable London dG and GBVI/WSA dG scores of −10.48 and −7.59 kcal/mol, respectively, compared with −9.58 and −6.32 kcal/mol for compound **1**. More importantly, the difference persisted beyond the initial docking poses. During the 100 ns simulations, the complex with compound **7** remained within a relatively stable conformational range, whereas the complex with compound **1** showed a gradual increase in RMSD after approximately 45 ns. The calculated MM/PBSA binding free energy was also more favorable for compound **7** than for compound **1**, at −116.4 ± 7.9 versus −88.3 ± 8.7 kJ/mol. Taken together, these findings suggest that the difference in α-glucosidase inhibition is not explained merely by a more favorable static docking score [10], but also by more persistent accommodation of compound **7** within the binding pocket. The resorcinol hydroxyl groups may support polar anchoring near the catalytic region, whereas the C17:1 chain extends into a hydrophobic channel and provides additional van der Waals contacts [21]. This interpretation remains a computational model, but it is internally consistent with the experimental activity order.

The AChE inhibitory activity was considerably weaker. Compound **7** was the only detectable inhibitor, with an IC_50_ value of 14.75 ± 1.07 μg/mL, whereas donepezil gave an IC_50_ of 0.028 ± 0.002 μg/mL. Compound **7** was therefore several hundred-fold less potent than the reference inhibitor on a mass basis and should not be regarded as a strong AChE inhibitor. Its activity is nevertheless supported by a plausible binding mode [15]. Similar integrated computational approaches combining molecular docking and MD simulations have been applied to evaluate phytochemical interactions with α-glucosidase and AChE [51,52]. The molecule extended along the catalytic gorge and interacted with residues associated with both the catalytic anionic and peripheral regions. The complex remained stable during the 100 ns simulation, and its calculated binding free energy was −105 ± 9 kJ/mol. These results indicate that the measured inhibition is compatible with sustained occupation of the gorge, but they do not imply an affinity comparable to that of donepezil. The substantial potency difference likely reflects the more limited aromatic and electrostatic interaction network of compound **7** despite its favorable hydrophobic accommodation [25].

The DPPH assay provided further evidence that the phenolic structure of compound **7** contributes to its activity. Its IC_50_ value of 10.04 ± 1.05 μg/mL was close to that of quercetin under the same conditions, whereas compounds **1** and **6** showed weaker but measurable activity, with IC_50_ values of 36.01 ± 2.19 and 35.19 ± 1.50 μg/mL, respectively. Alkylresorcinols have previously been described as membrane-associated antioxidants whose behavior depends partly on their amphiphilic structure and the length of the alkyl chain [53]. In the present chemical assay, the two phenolic hydroxyl groups of compound **7** provide the most direct explanation for radical-scavenging, while the lipophilic chain may influence solubility and local concentration. However, the DPPH assay reflects a simplified chemical reaction and cannot by itself establish antioxidant effects in cells or in vivo.

The antimicrobial activity was weaker than the enzyme-inhibitory and radical-scavenging activities. Compound **7** inhibited *S. aureus* with an MIC of 64 μg/mL but was inactive against the other tested microorganisms at concentrations up to 256 μg/mL. This activity is substantially weaker than that of the reference antibiotic and is best classified as moderate [54]. The selectivity toward *S. aureus*, however, provided a basis for exploring a possible target rather than for proposing compound **7** as an antibacterial lead in its current form.

The substrate-similarity analysis produced a clear separation between enzymes involved in bacterial fatty-acid biosynthesis and the remaining evaluated targets. FabI received a final priority score of 74.95, followed by the FabF/FabH class at 69.25, whereas the next-ranked protein, tyrosyl-tRNA synthetase, scored only 29.90. This separation is chemically plausible because the long alkenyl substituent of compound **7** resembles the hydrophobic acyl portions of intermediates processed within the type II fatty-acid synthesis pathway. The target ranking should nevertheless be interpreted as a prioritization procedure rather than direct evidence of inhibition.

Docking provided additional, although still indirect, support for FabI. Compound **7** fitted into the hydrophobic channels of FabI, FabF and FabH, and the London dG values were similar among the three proteins. GBVI/WSA rescoring showed only a modest preference for FabI, with values of −8.35, −8.13 and −7.46 kcal/mol for FabI, FabH and FabF, respectively. The approximately 0.2 kcal/mol difference between FabI and FabH is too small to establish target selectivity from docking energy alone. FabI is nevertheless the more plausible candidate when the rescoring result is considered together with its highest substrate-similarity rank, the availability of a native *S. aureus* structure and the position of compound **7** adjacent to NADP within the enoyl-substrate channel (Figure 6A).

FabI is a validated antistaphylococcal target, and the crystal structure used in the present analysis contains the selective inhibitor AFN-1252 bound together with NADP [44]. Biochemical, genetic and structural investigations have previously confirmed FabI inhibition as the mechanism of AFN-1252, which exhibits markedly stronger antistaphylococcal activity than compound **7**. The resemblance between the predicted position of compound **7** and the NADP-adjacent binding region is therefore mechanistically relevant, but the two compounds should not be considered equivalent. AFN-1252 is a highly optimized inhibitor with extensive target-specific interactions [44], whereas compound **7** appears to rely predominantly on accommodation of its hydrophobic chain and limited anchoring by the resorcinol group. This difference is compatible with the moderate MIC observed for compound **7**.

FabF and FabH remain reasonable alternative targets because both β-ketoacyl-ACP synthases contain hydrophobic channels for acyl substrates: FabH initiates FASII by condensing an acyl-CoA primer with malonyl-ACP, whereas FabF participates in subsequent chain elongation [43,45,46]. However, these results require particular caution because the FabF and FabH structures used here are *E. coli* homologous templates rather than native *S. aureus* structures. Their docking results should therefore be regarded mainly as structural support for possible accommodation of **7** within β-ketoacyl synthase pockets. Direct inhibition assays against *S. aureus* FabI, FabF, and FabH would be required to determine whether FASII inhibition contributes to the observed antibacterial activity.

The SwissADME analysis further highlighted important differences in developability between the two active compounds of *A. sylvestris*. Compound **1** showed a comparatively favorable oral drug-likeness profile, whereas compound **7** was limited by high lipophilicity, poor predicted gastrointestinal absorption, and increased molecular flexibility. The absence of predicted BBB permeation suggests limited passive CNS exposure for both compounds, while the predicted inhibition of CYP1A2, CYP2C19, and CYP2D6 by compound **7** indicates a potential risk of metabolism-related drug–drug interactions. These findings do not diminish the observed biological activities of compound **7**, but indicate that further optimization or formulation strategies would likely be required before considering translational development.

The present study has several limitations. The biological activities were evaluated mainly using enzyme-based and chemical assays without cellular or in vivo validation. In addition, the proposed protein targets and binding mechanisms were inferred from molecular docking and molecular dynamics simulations and therefore require direct biochemical confirmation.

## 5. Conclusions

This study identified eight known constituents from *A. sylvestris* leaves and evaluated their α-glucosidase inhibitory, acetylcholinesterase inhibitory, DPPH radical-scavenging, and antimicrobial activities. Among them, 5-(8Z-heptadecenyl)resorcinol (**7**) showed the most favorable overall biological profile, with the strongest α-glucosidase inhibition, moderate AChE inhibition, strong DPPH radical-scavenging activity, and moderate antibacterial activity against *S. aureus*. Molecular docking, 100 ns molecular dynamics simulations, and MM/PBSA analysis provided complementary structural insights into its interactions with α-glucosidase and AChE, while target-prioritization and comparative docking suggested FabI as a plausible antibacterial target. These findings expand the phytochemical and biological characterization of *A. sylvestris* and support further experimental investigation of compound **7**.

## Figures and Tables

**Figure 1:**
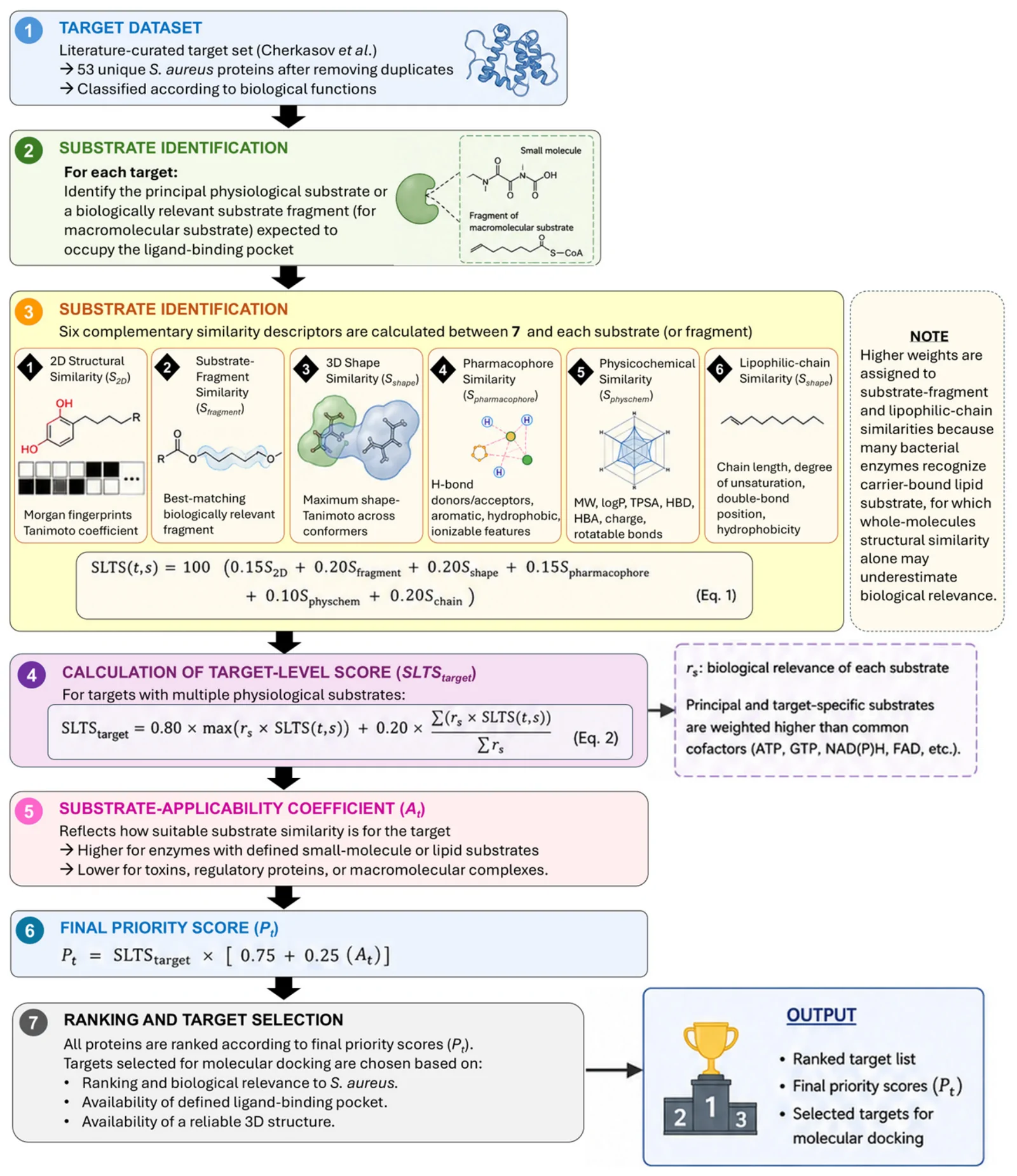
Workflow of the substrate-similarity-based target-prioritization framework developed for compound **7** against *S. aureus* [19].

**Figure 2:**
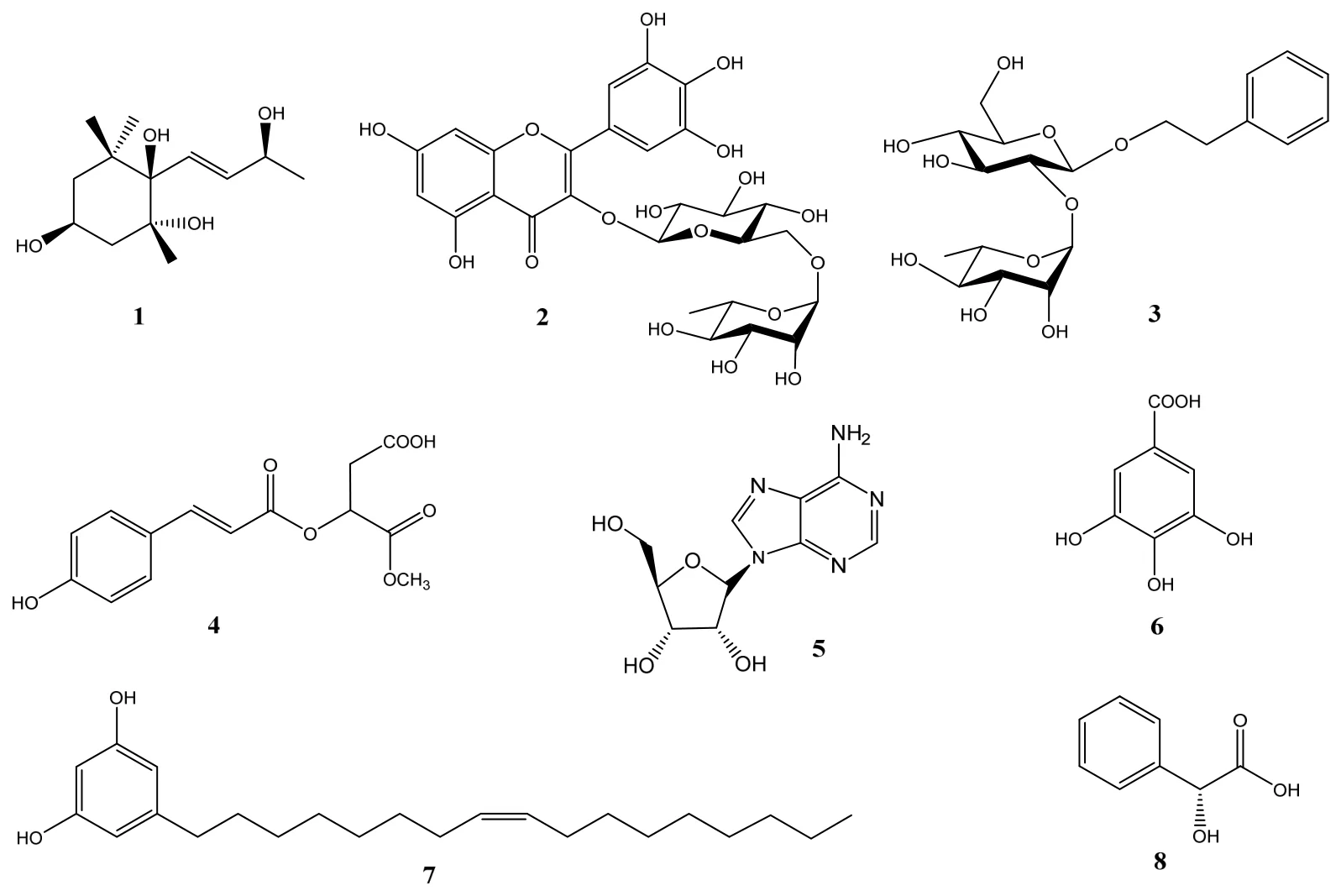
Chemical structure of compounds **1**–**8**.

**Figure 3:**
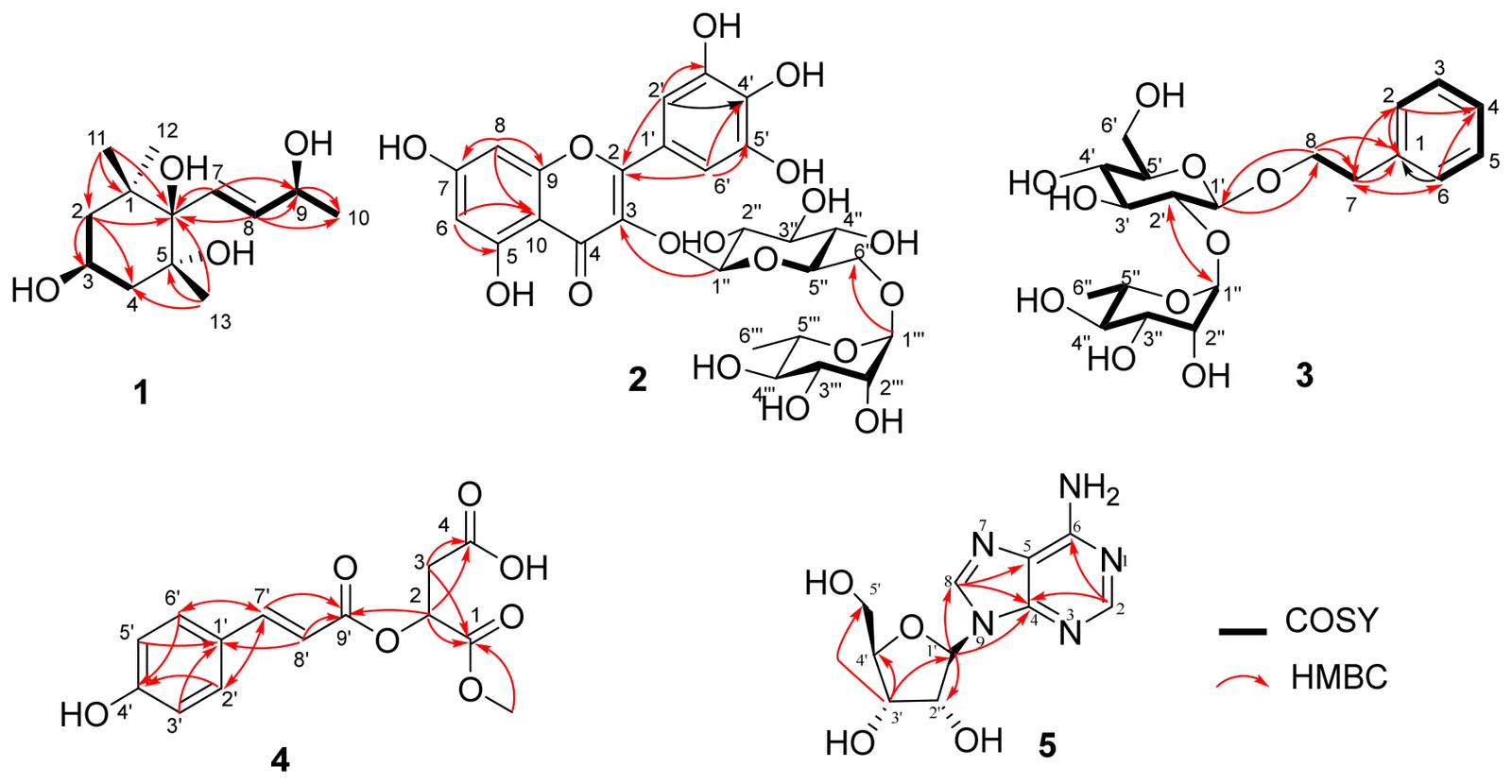
Key COSY and HMBC correlations of **1**–**5**.

**Figure 4:**
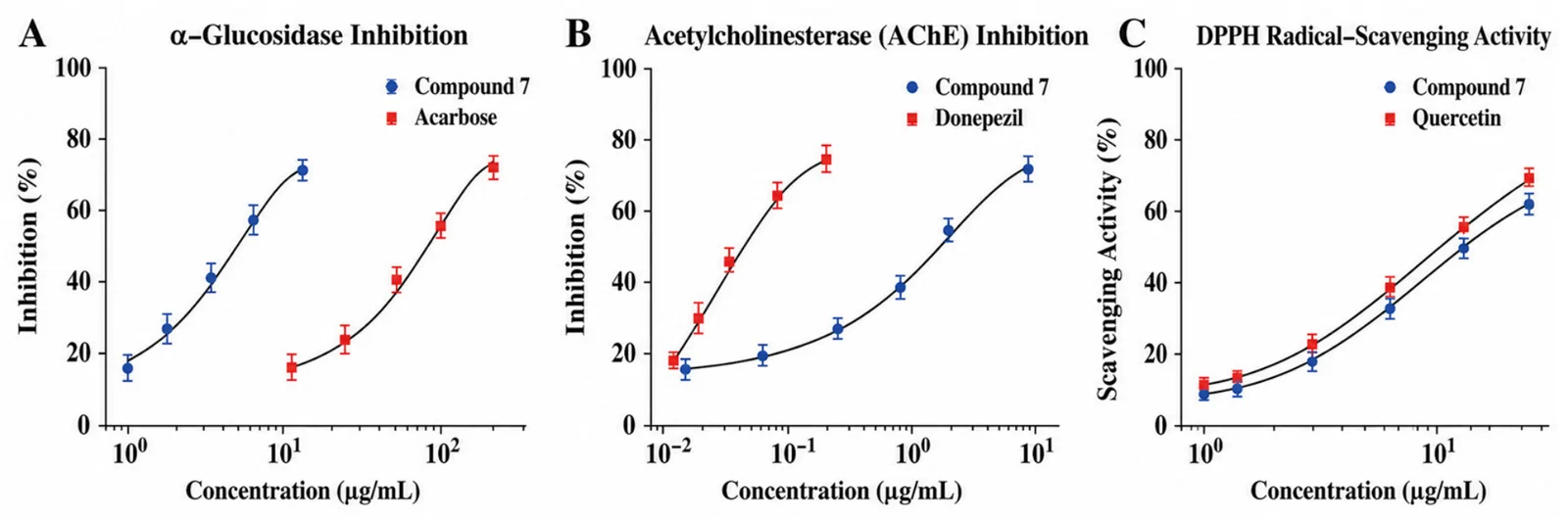
Concentration–response curves of (**A**) α-glucosidase inhibition by **7** and acarbose, (**B**) AChE inhibition by **7** and donepezil, and (**C**) DPPH radical-scavenging activity of **7** and quercetin. Data are expressed as mean ± SD (n = 3).

**Figure 5:**
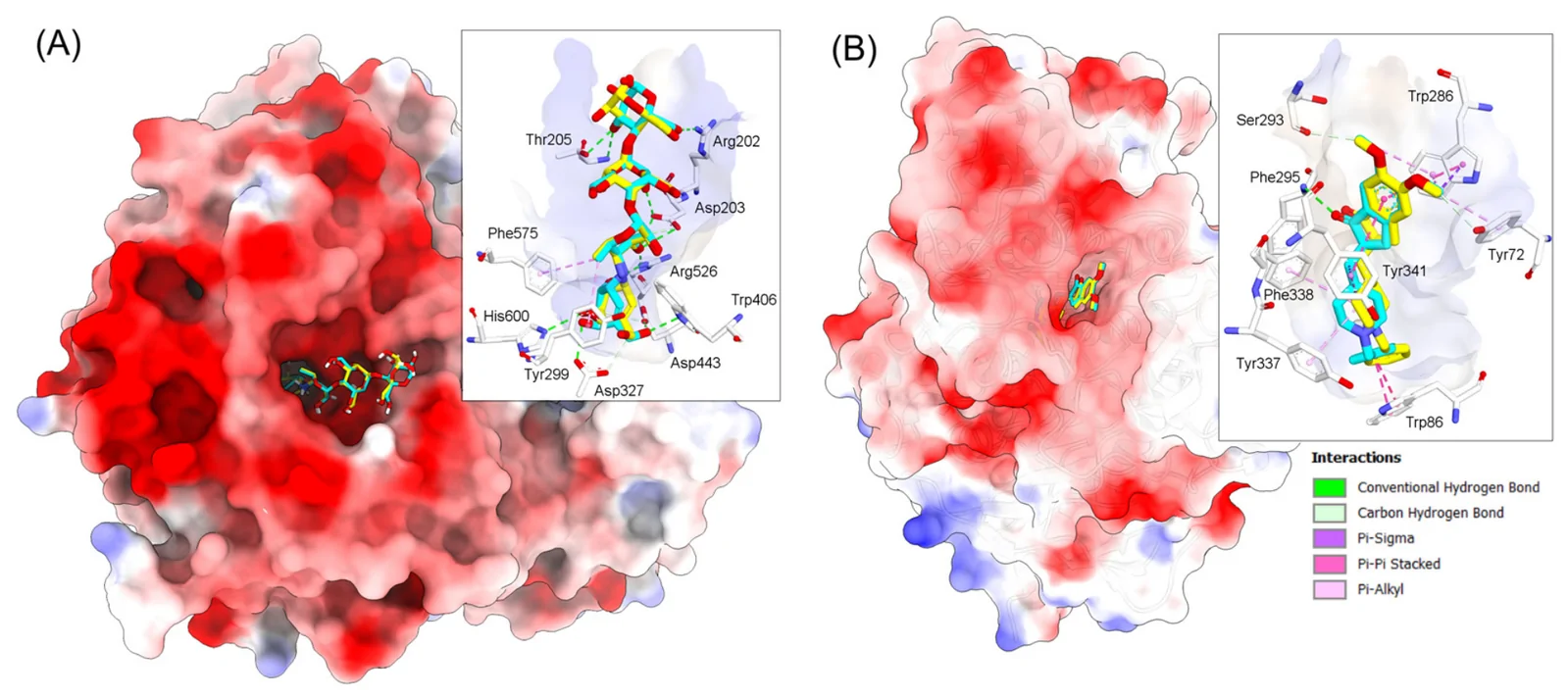
Redocking results of the co-crystallized ligands into the active sites of (**A**) human α-glucosidase (PDB ID: 2QMJ) [21] and (**B**) human acetylcholinesterase (PDB ID: 4EY7) [22]. Insets show the superimposed crystallographic (cyan) and redocked (yellow) ligand conformations together with their protein–ligand interactions.

**Figure 6:**
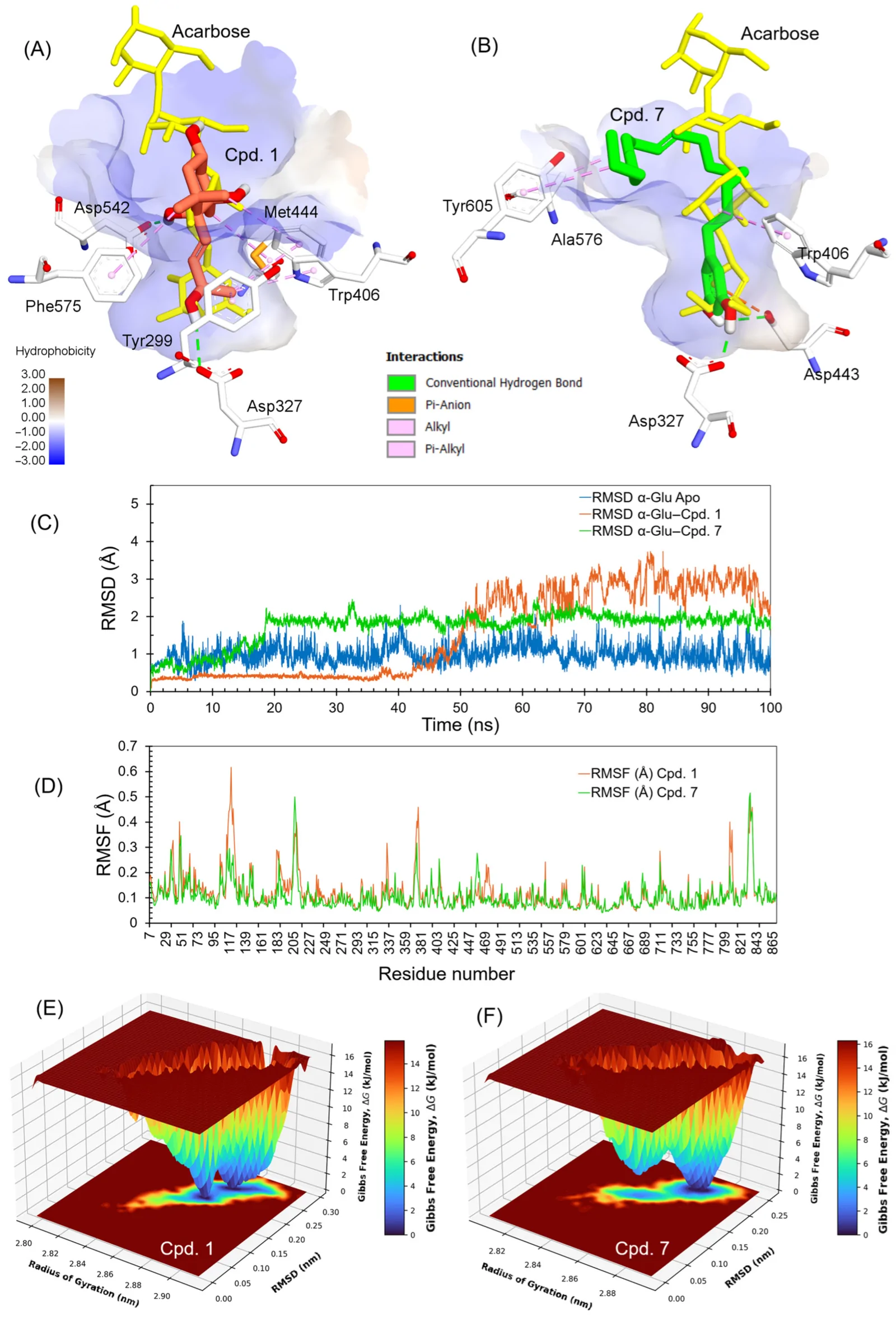
Molecular docking and molecular dynamics simulation of compounds **1** and **7** with human α-glucosidase. (**A**,**B**) Predicted binding modes compared to acarbose. (**C**) RMSD trajectories of the apo protein and ligand-bound complexes. (**D**) RMSF profiles. (**E**,**F**) Free-energy landscapes (FELs) of the complexes with compounds **1** and **7**, respectively.

**Figure 7:**
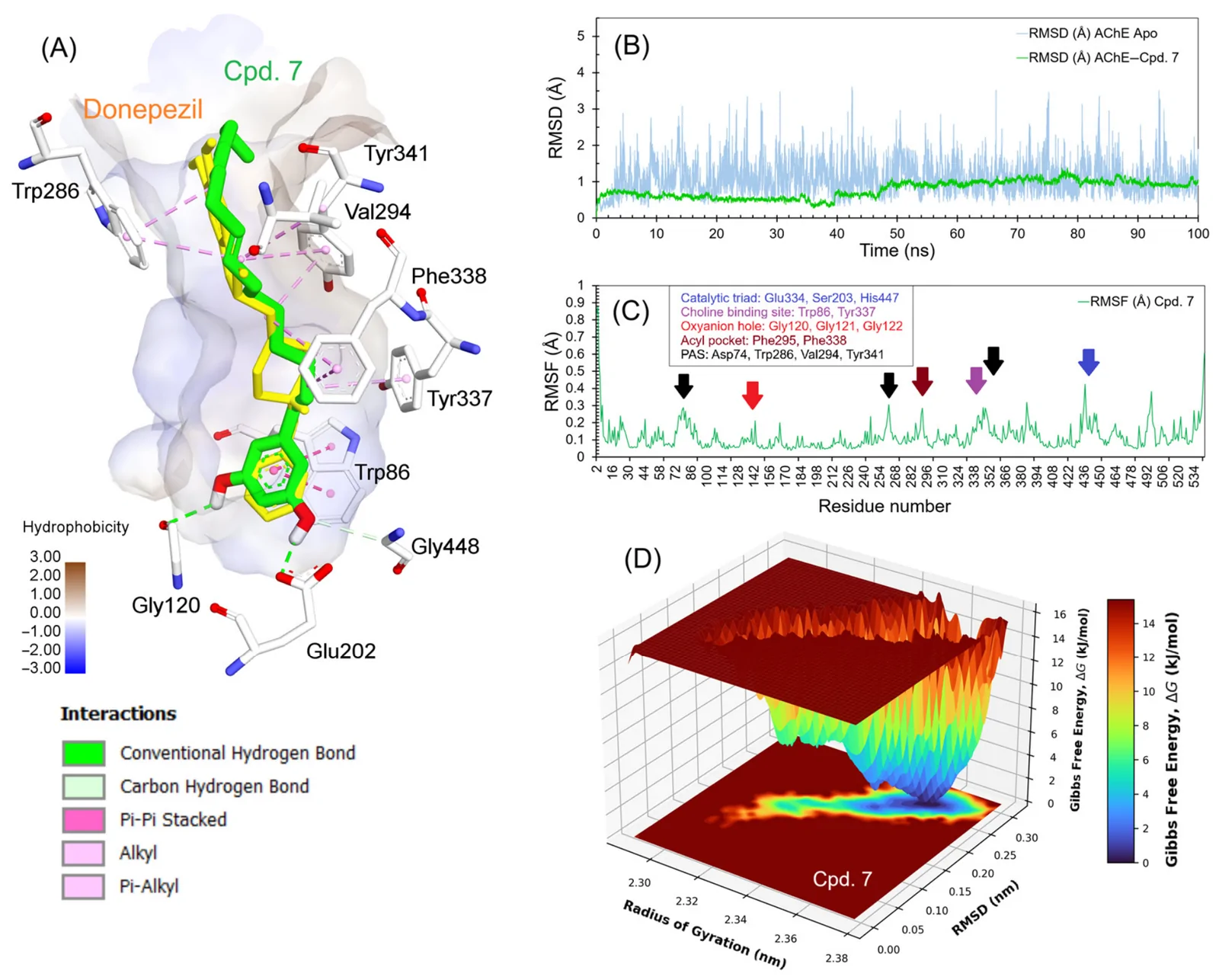
Molecular docking and molecular dynamics simulation of compound **7** in complex with human acetylcholinesterase (PDB ID: 4EY7) [22]. (**A**) Predicted binding pose superimposed with the co-crystallized ligand donepezil. (**B**) RMSD profiles of the apo protein and ligand-bound complex. (**C**) RMSF profile with the principal functional subsites of the catalytic gorge indicated. (**D**) FEL of the AChE–compound **7** complex.

**Figure 8:**
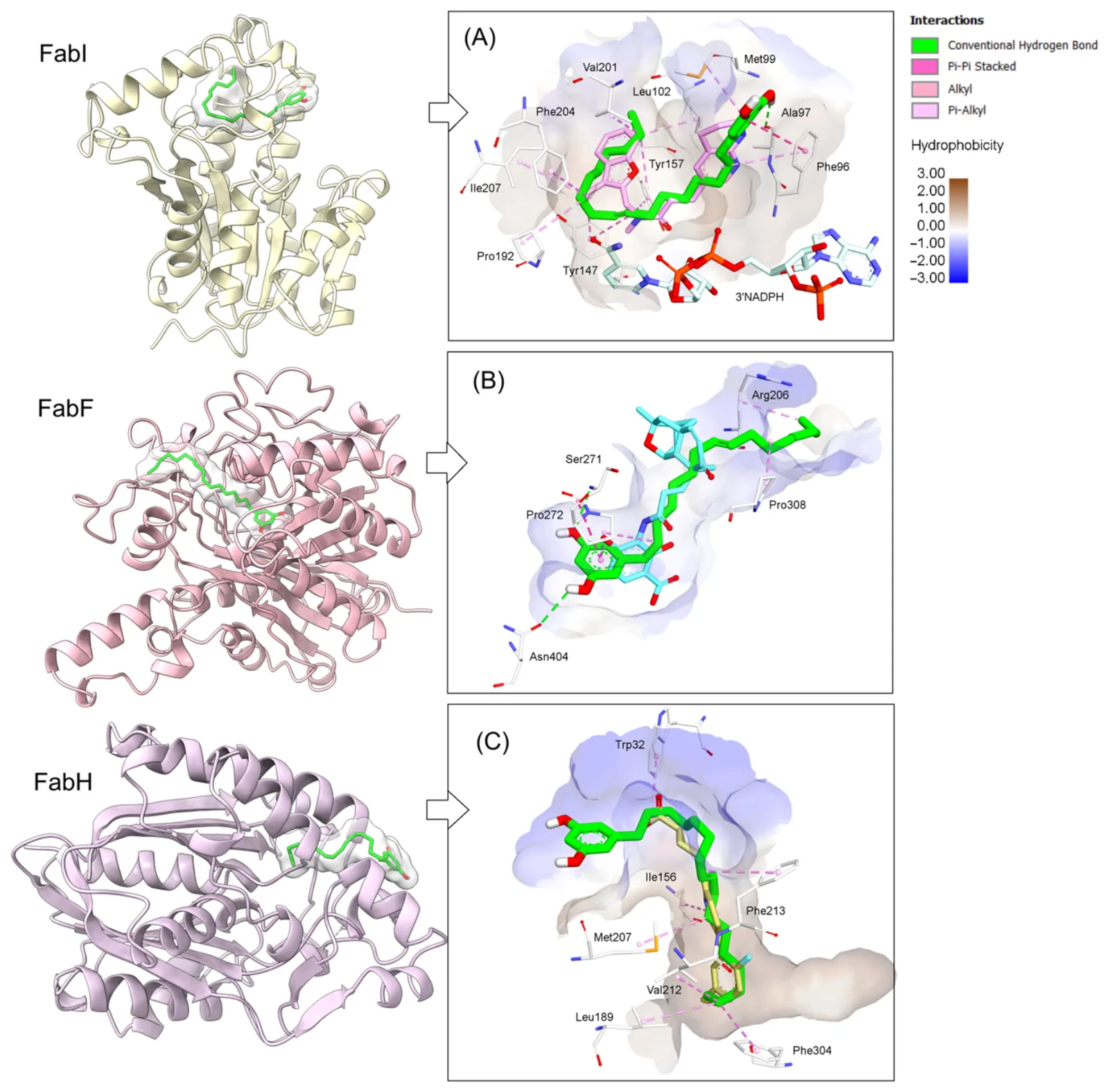
Comparative molecular docking of compound **7** with cocrystal ligands in the active sites of fatty-acid-biosynthesis targets. (**A**) Enoyl-acyl carrier protein reductase (FabI, PDB ID: 4FS3) [44]. (**B**) 3-Oxoacyl-acyl carrier protein synthase II (FabF, PDB ID: 2GFX) [45]. (**C**) 3-Oxoacyl-acyl carrier protein synthase III (FabH, PDB ID: 5BNR) [46].

**Figure 9:**
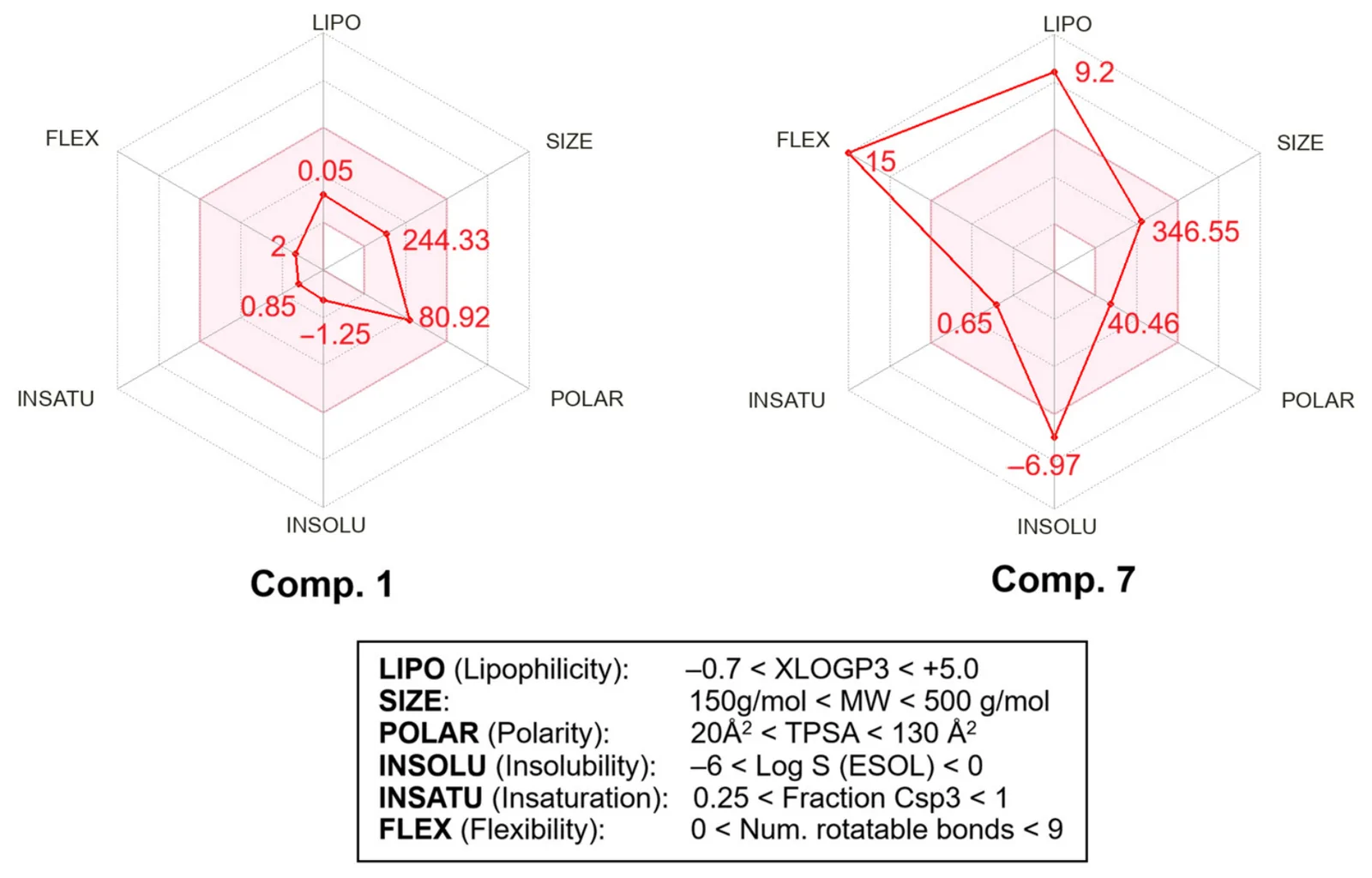
SwissADME-predicted physicochemical profiles and bioavailability radar plots of compounds **1** and **7**.

**Table 1:** In vitro biological activities of compounds isolated from *A. sylvestris* leaves.

Compound	Amount Isolated (mg)	α-Glucosidase	AChE	DPPH
		IC_50_ (µg/mL) ^1^		
**1**	5	16.79 ± 0.84	>256	36.01 ± 2.19
**2**	12	>256	>256	>256
**3**	3	52.00 ± 1.25	>256	>256
**4**	5	125.09 ± 2.74	>256	108.14 ± 8.29
**5**	9	>256	>256	62.55 ± 3.23
**6**	15	230.40 ± 5.58	>256	35.19 ± 1.50
**7**	6	7.56 ± 0.34	14.75 ± 1.07	10.04 ± 1.05
**8**	15	>256	>256	67.00 ± 4.08
Acarbose		151.09 ± 6.22	–	–
Donepezil		–	0.028 ± 0.002	–
Quercetin		–	–	8.65 ± 0.68

^1^ Values are expressed as mean ± SD (n = 3).

**Table 2:** Docking scores and key interactions of selected compounds and reference ligands with α-glucosidase and AChE.

Target ^1^	Ligand	London dG ^2^ (kcal/mol)	GBVI/WSA ^2^ dG (kcal/mol)	Key Interacting Residues
α-Glucosidase	Acarbose	−11.60	−9.90	Asp203, Asp327, Asp443, Asp542, Arg526, Tyr299, Phe575, His600
	Compound **1**	−9.58	−6.32	Asp327, Asp542, Tyr299, Phe575, Met444, Trp406
	Compound **7**	−10.48	−7.59	Asp327, Asp443, Trp406, Tyr605, Ala576
AChE	Donepezil	−12.30	−9.20	Trp86, Trp286, Phe295, Tyr337, Phe338, Tyr341
	Compound **7**	−10.19	−8.13	Trp86, Tyr337, Phe338, Trp286, Val294, Tyr341, Gly120, Glu202, Gly448

^1^ Protein data from PDB, including α-Glucosidase (ID: 2QMJ) [21] and AChE (ID: 4EY7) [22]; ^2^ Docking scores are reported in kcal/mol. More negative values indicate more favorable predicted binding. Reference-ligand values correspond to redocking of the co-crystallized ligands.

**Table 3:** Antimicrobial activities of compounds isolated from *A. sylvestris* leaves.

Compound	*S. aureus*	*B. subtilis*	*E. coli*	*P. aeruginosa*	*C. albicans*
	MIC (μg/mL) ^1^				
**1**	>256	>256	>256	>256	>256
**2**	>256	>256	>256	>256	>256
**3**	>256	>256	>256	>256	>256
**4**	>256	>256	>256	>256	>256
**5**	>256	>256	>256	>256	>256
**6**	>256	>256	>256	>256	>256
**7**	64	>256	>256	>256	>256
**8**	>256	>256	>256	>256	>256
Ampicillin	0.125	32	–	–	–
Cefotaxime	–	0.5	8	–	–
Nystatin	–	–	–	–	8

^1^ Value of MIC determined by the broth microdilution method.

**Table 4:** Highest-ranked potential *S. aureus* targets of **7** based on computational substrate-similarity scoring.

Target	Functional Pathway	*S_2D_*	*S_fragment_*	*S_shape_*	*S_pharmacophore_*	*S_physchem_*	*S_chain_*	*SLTS*_(*t*,*s*)_	Final Priority *
Enoyl-ACP reductase (FabI)	Fatty acid biosynthesis	0.43	0.81	0.82	0.68	0.73	0.92	74.95	70.27
3-Oxoacyl-ACP synthase class (FabF/FabH)	Fatty acid biosynthesis	0.39	0.74	0.77	0.58	0.69	0.88	69.25	64.92
Tyrosyl-tRNA synthetase	Aminoacyl-tRNA synthesis	0.22	0.39	0.43	0.36	0.42	0.03	29.90	29.90
Tryptophanyl-tRNA synthetase	Aminoacyl-tRNA synthesis	0.14	0.26	0.32	0.25	0.33	0.03	21.35	21.35
Signal peptidase Ib	Protein maturation and secretion	0.10	0.21	0.28	0.20	0.30	0.18	20.90	20.90
Anthranilate synthase component I	Aromatic amino acid biosynthesis	0.13	0.24	0.28	0.22	0.29	0.02	18.95	18.95
Methionyl-tRNA synthetase	Aminoacyl-tRNA synthesis	0.10	0.20	0.26	0.19	0.28	0.10	18.35	18.35
Leucyl-tRNA synthetase	Aminoacyl-tRNA synthesis	0.09	0.18	0.23	0.17	0.26	0.09	16.50	16.50
Acetyl-CoA carboxylase carboxyl transferase subunit α	Fatty acid biosynthesis	0.10	0.17	0.21	0.16	0.25	0.12	16.40	16.40
Isoleucyl-tRNA synthetase	Aminoacyl-tRNA synthesis	0.09	0.17	0.22	0.16	0.25	0.08	15.65	15.65

* Final priority was calculated as SLTS × [0.75 + 0.25 × substrate applicability]. Priority levels were assigned relative to the distribution of final priority scores among all 53 evaluated targets: very high, ≥95th percentile; high, 80th–<95th percentile; moderate, 60th–<80th percentile; low, 40th–<60th percentile; and very low, <40th percentile.

**Table 5:** Docking scores and key interactions of compound **7** and the co-crystallized ligands with the selected fatty-acid-biosynthesis targets.

Target	Ligand ^1^	London dG (kcal/mol)	GBVI/WSA dG (kcal/mol)	Key Interactions
FabI	Co-crystallized inhibitor	−12.19	−9.77	Interactions within the NADP-adjacent substrate pocket
	Compound **7**	−10.51	−8.35	H-bond: Tyr157; hydrophobic/π contacts: Phe96, Ala97, Met99, Leu102, Val201, Phe204, Ile207; positioned adjacent to NADP
FabF	Co-crystallized inhibitor	−10.47	−8.86	Interactions within the elongated acyl-substrate channel
	Compound **7**	−10.74	−7.46	H-bonds: Ser271, Asn404; hydrophobic/π contacts: Arg206, Pro272, Pro308
FabH	Co-crystallized inhibitor	−13.28	−10.38	Interactions within the acyl-substrate binding channel
	Compound **7**	−10.19	−8.13	π/alkyl contacts: Trp32, Ile156, Leu189, Met207, Val212, Phe213, Phe304

^1^ Co-crystallized inhibitors of FabI (PDB ID: 4FS3) [44], FabF (PDB ID: 2GFX) [45], and FabH (PDB ID: 5BNR) [46] are AFN-1252, platensimycin, and 1-{5-[2-fluoro-5-(hydroxymethyl)phenyl]pyridin-2-yl}piperidine-4-carboxylic acid, respectively.

**Table 6:** Selected SwissADME-predicted pharmacokinetic and drug-likeness properties of compounds **1** and **7**.

Property	Compound 1	Compound 7
GI absorption	High	Low
BBB permeation	No	No
P-gp substrate	Yes	No
CYP inhibition	None predicted	CYP1A2, CYP2C19, CYP2D6
Lipinski Rule of Five	0 violation	1 violation
Veber Rule	Yes	No
Bioavailability score	0.55	0.55
PAINS alerts	0	0

## Data Availability

The data supporting the findings of this study are available from the corresponding authors upon reasonable request.

## Supplementary Materials

The following supporting information can be downloaded at https://www.mdpi.com/article/10.3390/cimb48100986/s1.

## Abbreviations

The following abbreviations are used in this manuscript:

AChE	Acetylcholinesterase
DPPH	2,2-diphenyl-1-picrylhydrazyl
ATCC	American Type Culture Collection
SLTS	Substrate-likeness target score
RMSD	root mean square deviation
MD	Molecular dynamics
RMSF	Root mean square fluctuation
FEL	Free-energy landscape
IC_50_	Half-maximal inhibitory concentration
MIC	Minimum inhibitory concentration
SD	Standard deviation
